# Ovarian cancer-derived TGF-β1 induces cancer-associated adipocytes formation by activating SMAD3/TRIB3 pathway to establish pre-metastatic niche

**DOI:** 10.1038/s41419-024-07311-3

**Published:** 2024-12-24

**Authors:** Tian Gao, Jibin Li, Tianyi Cheng, Xingguo Wang, Mengqing Wang, Zhiyang Xu, Yang Mu, Xianli He, Jinliang Xing, Shujuan Liu

**Affiliations:** 1 Department of General Surgery, Tangdu Hospital, Fourth Military Medical University, Xi’an, 710038 China; 2 Department of Obstetrics and Gynaecology, Xijing Hospital, Fourth Military Medical University, Xi’an, 710032 China; 3 State Key Laboratory of Holistic Integrative Management of Gastrointestinal Cancers and Department of Physiology and Pathophysiology, Fourth Military Medical University, Xi’an, 710032 China

**Keywords:** Cancer microenvironment, Mechanisms of disease

## Abstract

Ovarian cancer (OC) is prone to adipose tissue metastasis. However, the underlying molecular mechanisms remain elusive. Here, we observed that omental adipocytes were induced into cancer-associated adipocytes (CAAs) by OC-derived TGF-β1 to establish a pre-metastatic niche (PMN) through collagen and fibronectin secretion. Mechanistically, OC-derived TGF-β1 binds to adipocyte membrane receptors and thus activates intracellular signaling by SMAD3 phosphorylation. The activation of TGF-β1/SMAD3 signaling pathway dedifferentiates adipocytes into CAAs by upregulating Tribbles homolog 3 (TRIB3), which suppresses the phosphorylation of CEBPβ. Additionally, CAAs secrete collagen I, collagen VI, and fibronectin to remodel the extracellular matrix and promote the adhesion of OC cells. Pharmacological inhibition of the TGF-β1/SMAD3 pathway significantly inhibits CAAs and PMN formation, thereby reducing the OC metastatic burden. Our findings indicate that the formation of CAAs and PMN in adipose tissues facilitates OC cell implantation and blocking the TGF-β1/SMAD3 signaling pathway could prevent OC omental metastasis.

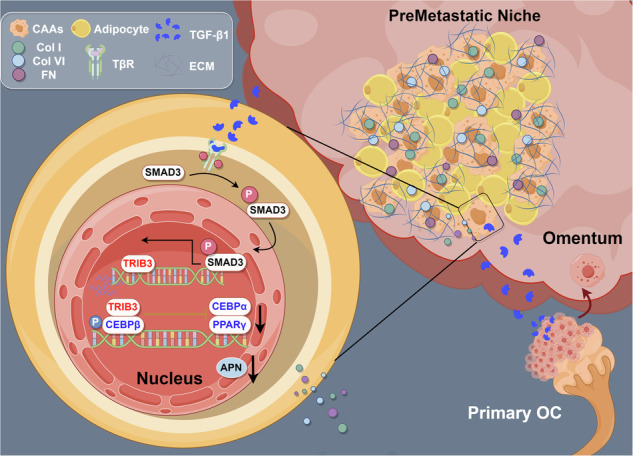

## Introduction

Ovarian cancer (OC) is the most lethal gynecological malignancy with a tendency to metastasize to adipose tissues (e.g., omentum and appendices epiploicae), especially in advanced stages [1]. Primary tumors selectively and actively modify the microenvironment of distant tissues, creating premetastatic niches (PMNs) that are conducive to the survival and growth of tumor cells before their recolonization [2–4]. Studies have shown that PMN formation results from the combined effects of tumor-derived factors and local cells at future metastatic sites [3]. Additionally, PMN formation contributes to organ-specific metastasis in certain tumor types [2, 4–7]. However, the mechanisms underlying PMN formation in OC-associated omental metastases are not completely clear.

A previous study has revealed that OC cells stimulate neutrophils to mobilize and extrude neutrophil extracellular traps to shape PMN in the omentum, promoting OC metastasis [8]. Additionally, tissue-resident macrophages promote the metastasis of OC cells to the omentum, whereas the specific depletion of CD163^+^ Tim4^+^ macrophages prevents the metastatic spread of OC [9]. The omentum is enriched by adipocytes. However, as the most abundant cell type in the omentum, the role of adipocytes in OC metastasis is not completely understood. Previous studies have shown that several types of cancer cells induce the normal adipocytes into cancer-associated adipocytes (CAAs), which are characterized by the dedifferentiated phenotype of adipocytes [10–14]. CAAs can increase the secretion of proinflammatory cytokines and extracellular matrix for cancer progression [11, 15, 16]. Thus, adipocytes are capable of reprogramming their microenvironment to form PMNs [11, 17, 18]. However, it is unknown whether CAAs exist in the omentum of patients with OC. A comprehensive investigation of CAAs in the adipose tissues is helpful for understanding the molecular mechanisms underlying OC omental metastasis.

In the present study, we demonstrate the presence of CAAs in the omentum before the initiation of OC metastasis. Mechanistically, OC-derived transforming growth factor-β 1 (TGF-β1) transforms normal adipocytes into CAAs by activating the SMAD family member 3 (SMAD3)/TRIB3 signaling pathway. Furthermore, CAAs contribute to PMN formation by remodeling the extracellular matrix via collagen and fibronectin secretion. Importantly, our findings show that pharmacological blockage of the TGF-β1/SMAD3 pathway markedly inhibited the induction of CAAs and the formation of PMN, thereby reducing OC metastatic burden.

## Results

### CAAs exist in the adipose tissues surrounding omental metastases and in the premetastatic omentum of patients and mice with OC

**Fig. 1:**
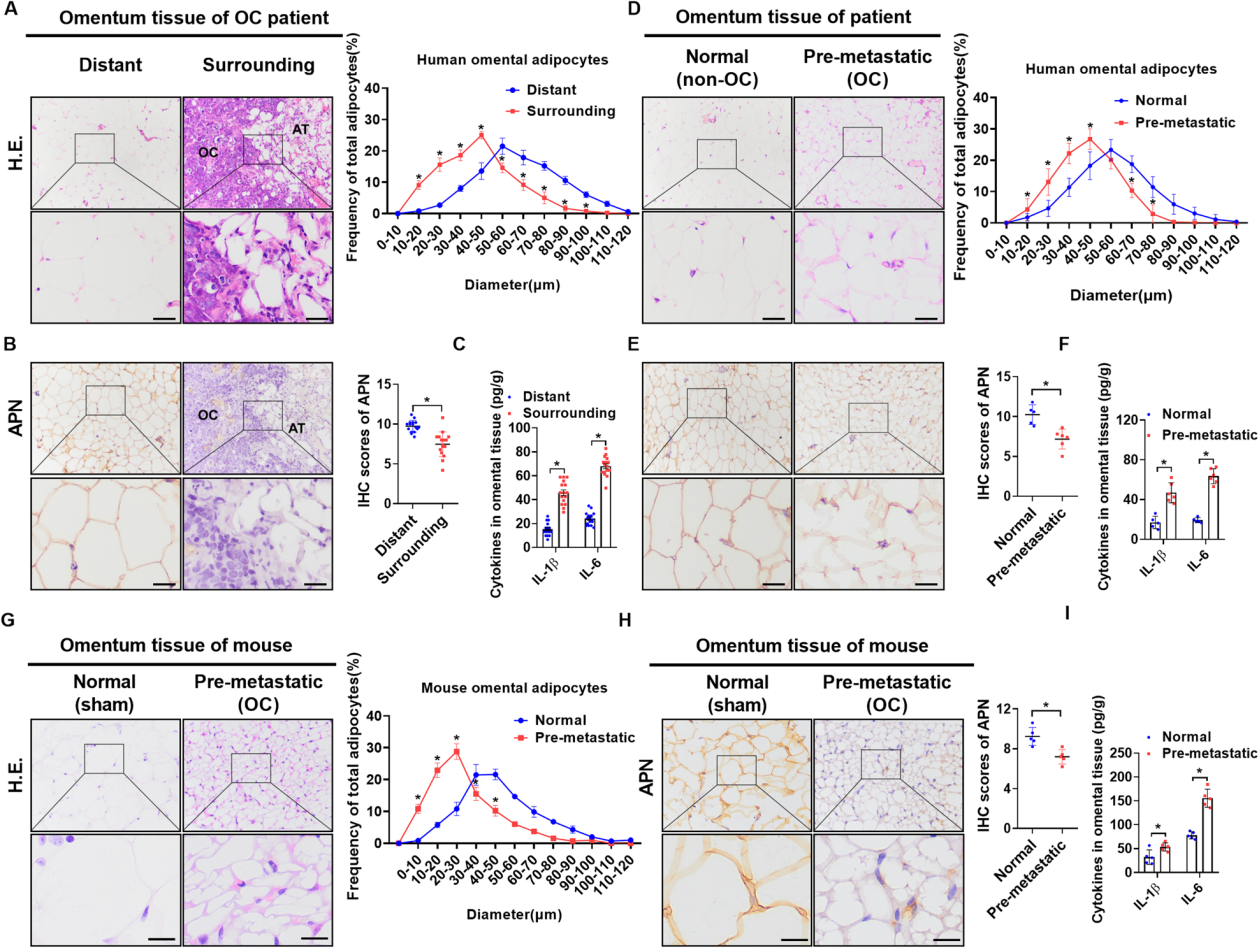
CAAs exist in the adipose tissues surrounding omental metastases and in the premetastatic omentum of patients and mice with OC. **A** Representative H.E. images (left panel) and distribution of adipocyte diameter (µm) (right panel) in adipose tissues surrounding or distant from omental metastases from 16 OC patients (Scale bar = 25 µm). OC ovarian cancer, AT adipose tissue. **B** Representative IHC staining images (left panel) and scores of APN in adipose tissues surrounding or distant from omental metastases of OC patients (*n* = 16; Scale bar = 25 µm). OC ovarian cancer, AT adipose tissue. **C** The content of cytokines in adipose tissues surrounding or distant from omental metastases was measured by ELISA (*n* = 16 per group). **D** Representative H.E. images (left panel) and distribution of adipocyte diameter (µm) (right panel) in normal (*n* = 5) or pre-metastatic (*n* = 6) omental adipose tissues (Scale bar = 25 µm). **E** Representative IHC staining images (left panel) and scores of APN in normal or pre-metastatic adipose tissues (normal: *n* = 5, pre-metastatic: *n* = 6; Scale bar = 25 µm). **F** The content of cytokines in adipose tissues was measured by ELISA (normal: *n* = 5, pre-metastatic: *n* = 6). **G** Representative H.E. images (left panel) and distribution of adipocyte diameter (µm) (right panel) in normal or pre-metastatic adipose tissues of mice (Scale bar = 10 µm; *n* = 5 per group). **H** Representative IHC staining images (left panel) and scores of APN in normal (*n* = 5 per group) or pre-metastatic (*n* = 5 per group) adipose tissues of mice (Scale bar = 15 µm). **I** The content of cytokines in normal or pre-metastatic adipose tissues of mice was measured by ELISA (*n* = 5 per group). Data were expressed as mean ± SEM. Student’s *t* test was used for analysis of the data in **B**, **C**, **E**, **F**, **H** and **I**. Two-way ANOVA with Bonferroni’s post hoc test was used for analysis of the data in **A**, **D** and **G**. *p < 0.05.

To investigate the presence of CAAs in the omentum of patients with OC, adipose tissues surrounding (within 1 cm of the edge) and distant from (more than 2 cm from the edge) omental metastases were collected from 16 patients with advanced OC. Histological analyses revealed a significantly reduced adipocyte diameter and expression of adiponectin (APN), a marker representing the terminal differentiation of adipocytes (Fig. 1A, B), in the adipose tissues surrounding OC omental metastases compared to paired adipose tissues distant from OC metastases. ELISA showed higher concentrations of IL-1β and IL-6 in the surrounding adipose tissues when compared with those in paired tissues (Fig. 1C). Similar results were found in omental adipose tissues from five non-cancer patients and six patients with early-stage OC, without macroscopic metastases, indicating the existence of CAAs in the pre-metastatic omental adipose tissues of patients with early-stage OC (Fig. 1D–F). To further validate the presence of CAAs in the premetastatic omentum, orthotopic models were generated by i.b. injection of mouse OC cell line ID8 into C57BL/6 mice (Supplementary Fig. 1A) to mimic metastatic tropism in patients with OC. Similar results were obtained, indicating the presence of CAAs in the premetastatic omental adipose tissues of early-stage OC mice (Fig. 1G–I).

### Conditioned medium from OC cells dedifferentiates adipocytes into CAAs in vitro and in vivo

**Fig. 2:**
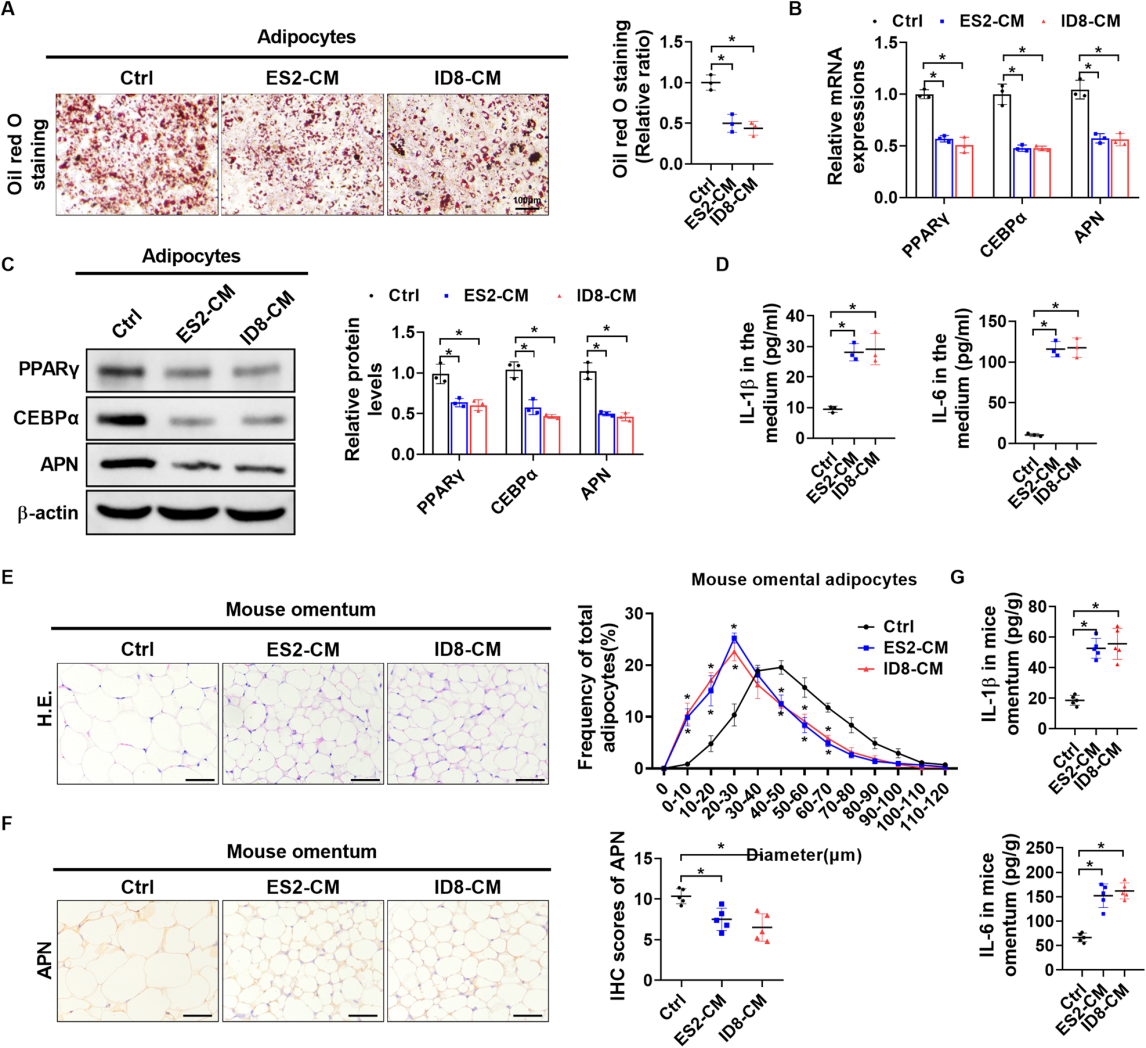
Conditioned medium from OC cells dedifferentiates adipocytes into CAAs in vitro and in vivo. **A** Oil red O staining of adipocytes with treatment as indicated (*n* = 3 independent experiments, Scale bar = 100 µm). **B**, **C** Quantitative RT-PCR and western blot analysis for mRNA and protein expression levels of PPARγ, CEBPα and APN in adipocytes with treatment as indicated (*n* = 3 independent experiments). **D** ELISA analysis for concentrations of IL-1β and IL-6 in the medium of adipocytes with treatment as indicated (*n* = 3 independent experiments). **E**, **F** Female C57BL/6 mice were injected intraperitoneally with 300 mL OC-derived CM (*n* = 5 per group) or DMEM (*n* = 5 per group) daily over the next 3 weeks, respectively. Representative H.E. images (left panel) and distribution of adipocyte diameter (µm) (right panel) of adipose tissues of mice with indicated treatments (Scale bar = 60 µm). **G** The contents of cytokines in adipose tissues of mice with indicated treatments were measured by ELISA. Data were expressed as mean ± SEM. Student’s *t* test was used for analysis of the data in **A**–**D** and **G**. Two-way ANOVA with Bonferroni’s post hoc test was used for analysis of the data in **F**. *p < 0.05.

To determine whether the transformation of adipocytes into CAAs was induced by OC-derived factors, mature adipocytes induced from 3T3-L1 cells were cultured in conditioned medium (CM) collected from mouse and human OC cell lines ID8 (ID8-CM) and ES2 (ES2-CM) (Supplementary Fig. 2A, B). Oil Red O staining analysis showed a decrease in lipid droplet content in adipocytes cultured with OC-derived CM for 48 h (Fig. 2A). Peroxisome proliferator-activated receptor gamma (PPARγ) and CCAAT-enhanced binding protein alpha (CEBPα) are the master regulators of adipocyte differentiation. Both qRT-PCR and western blot analyses demonstrated the down-regulation of PPARγ, CEBPα, and adipocytes terminal differentiation marker APN in co-cultured adipocytes at both mRNA and protein levels (Fig. 2B, C). ELISA also revealed remarkably increased IL-1β and IL-6 contents in the medium of co-cultured adipocytes (Fig. 2D).

To further explore whether key OC-derived molecules also induce adipocyte differentiation into CAAs in vivo, female C57BL/6 mice were intraperitoneally injected with OC-derived CM or DMEM. H.E. and Immunohistochemical (IHC) staining assays showed that the diameter of adipocytes and APN expression were decreased in mouse omental adipocytes treated with OC-derived CM compared to the control group (Fig. 2E, F). The contents of IL-6 and IL-1β were significantly increased in CM-treated mouse omental adipose tissues (Fig. 2G). These results indicate that OC-derived CM induces the dedifferentiation of adipocytes into CAAs in vitro and in vivo.

### Conditioned medium from OC cells induces CAAs formation via upregulating TRIB3 to suppress the phosphorylation of CEBPβ

**Fig. 3:**
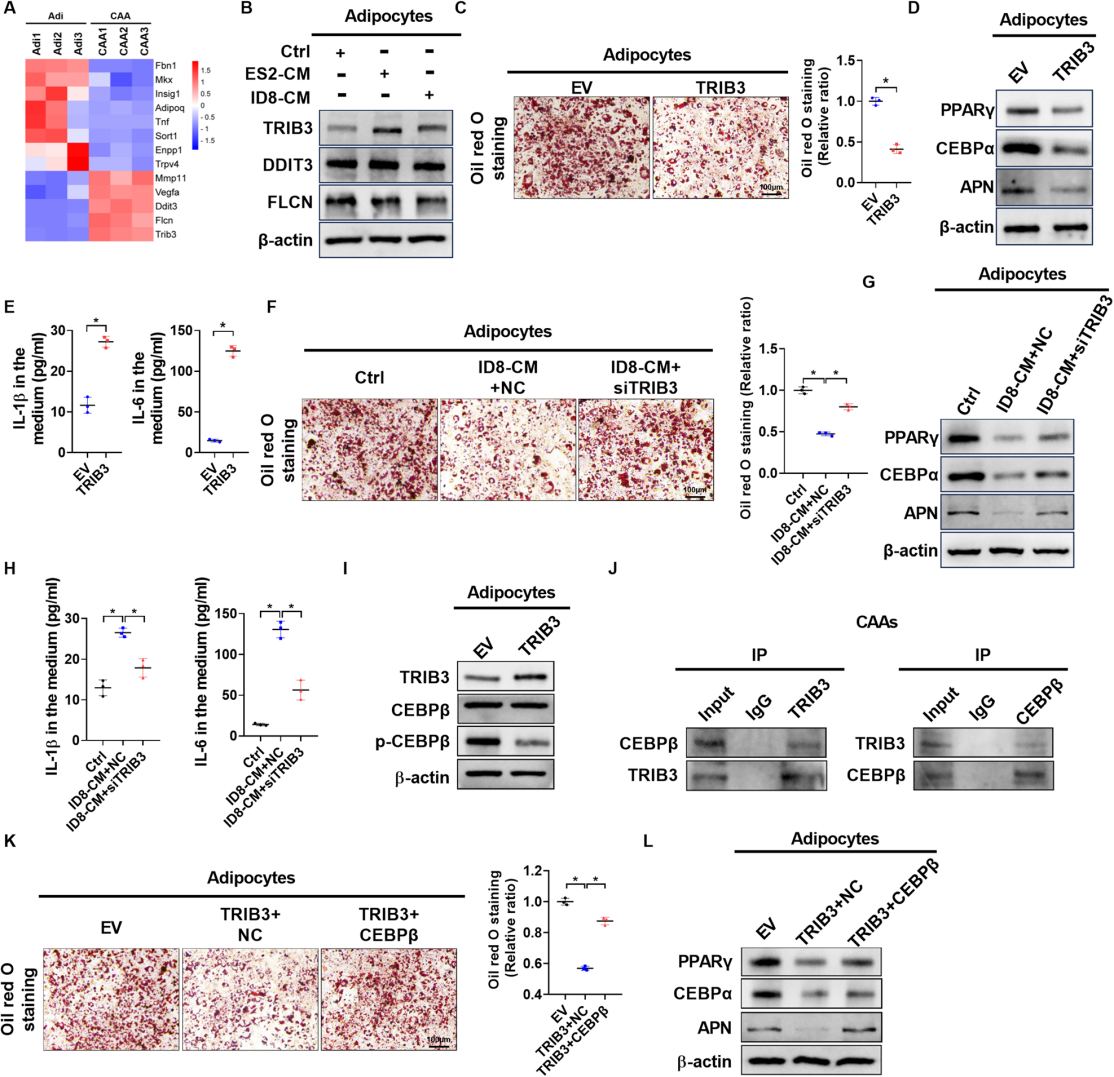
Conditioned medium from OC cells induces CAAs formation via upregulating TRIB3 to suppress phosphorylation of CEBPβ. **A** Heatmap of differentially expressed genes (DEGs) that are involved in negative regulation of adipocyte differentiation between control adipocytes and CAAs. **B** Western blot analysis for protein expression levels of TRIB3, DDIT3, and FLCN in adipocytes with treatment as indicated. **C** Oil red O staining of adipocytes with treatment as indicated. **D** Western blot analysis for protein expression levels of PPARγ, CEBPα and APN in adipocytes with treatment as indicated. **E** ELISA analysis for concentrations of IL-1β and IL-6 in the medium of adipocytes with treatment as indicated. **F** Oil red O staining of adipocytes with treatment as indicated. **G** Western blot analysis for protein expression levels of PPARγ, CEBPα and APN in adipocytes with treatment as indicated. **H** ELISA analysis for concentrations of IL-1β and IL-6 in the medium of adipocytes with treatment as indicated. **I** Western blot analysis for protein expression levels of TRIB3, CEBPβ, and p-CEBPβ in adipocytes with treatment as indicated. **J** Co-IP assay was performed with anti-TRIB3 or anti-CEBPβ antibodies using extracts prepared from CAAs. The presence of TRIB3 or CEBPβ in IPs was evaluated by immunoblotting (WB). **K** Oil red O staining of adipocytes with treatment as indicated. **L** Western blot analysis for protein expression levels of PPARγ, CEBPα and APN in adipocytes with treatment as indicated. Scale bar = 100 µm. Data were expressed as mean ± SEM of three independent experiments. Student’s t test was used for analysis of the data in **C**, **E**, **F**, **H** and **K**. *p < 0.05.

To explore the molecular mechanisms underlying CAA formation, differently expressed genes (DEGs) between CAAs and control adipocytes were analyzed using RNA-seq. Consistent with the above results, the mRNA expressions of PPARγ, CEBPα, and APN were significantly decreased in CAAs compared to control adipocytes (Supplementary Fig. 3A). Notably, five crucial factors involved in the negative regulation of adipocyte differentiation were significantly upregulated (Fig. 3A). Western blot analyses showed that only the protein expression level of TRIB3 was significantly upregulated in CAAs compared to control adipocytes (Fig. 3B, Supplementary Fig. 3B). Knocking down or overexpressing TRIB3 in adipocytes confirmed its role in the formation of CAAs, as verified by qRT-PCR and western blot analyses (Supplementary Fig. 3C–F). The content of lipid droplets and expression levels of PPARγ, CEBPα, and APN were dramatically decreased in adipocytes upon TRIB3 overexpression, while the content of IL-1β and IL-6 in the culture medium was significantly increased (Fig. 3C–E and Supplementary Fig. 3G). In addition, *TRIB3* knockdown reversed the dedifferentiation of adipocytes induced by OC-derived CM (Fig. 3F–H, Supplementary Fig. 3H). These findings demonstrate that CM from OC cells induces the formation of CAAs by upregulating TRIB3 expression.

A previous study has reported that TRIB3 overexpression inhibits the differentiation of 3T3-L1 cells into mature adipocytes by interacting with the transcription factor CEBPβ to suppress the expression of PPARγ and CEBPα. TRIB3 also blocks extracellular signal-regulated kinase activity to prevent the phosphorylation of regulatory sites on CEBPβ [19]. Thus, we hypothesize that the overexpression of TRIB3 dedifferentiates mature adipocytes into CAAs by interacting with CEBPβ to inhibit its activity. Western blot analyses showed that TRIB3 overexpression significantly decreased the phosphorylation of CEBPβ in adipocytes (Fig. 3I). Moreover, Co-immunoprecipitation (Co-IP) assays confirmed the direct interaction between TRIB3 and CEBPβ (Fig. 3J). Expectedly, CEBPβ overexpression reversed TRIB3-induced decrease of lipid droplets and dedifferentiation of adipocytes (Fig. 3K, L, and Supplementary Fig. 3I), indicating that TRIB3 dedifferentiates adipocytes into CAAs by interacting with CEBPβ to suppress its phosphorylation.

### OC-derived TGF-β1 contributes to CAAs formation by activating the SMAD3/TRIB3 signaling pathway

**Fig. 4:**
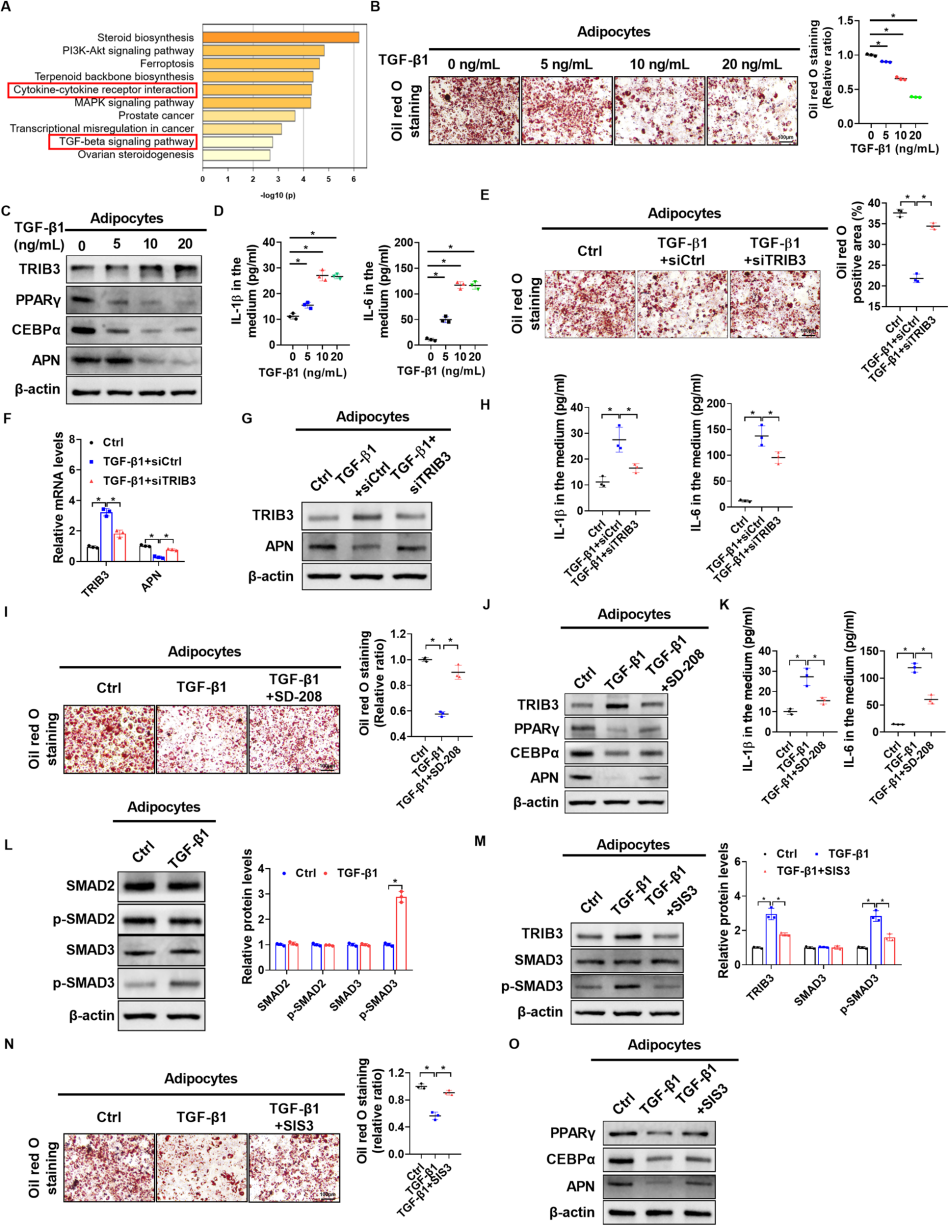
OC-derived TGF-β1 contributes to CAAs formation by activating the SMAD3/TRIB3 signaling pathway. **A** The KEGG pathways enriched by Metascape (top 200 DEGs between control adipocytes and CAAs). **B** Oil red O staining assays of adipocytes with treatment as indicated. **C** Western blot analysis for protein expression levels of PPARγ, CEBPα and APN in adipocytes with treatment as indicated. **D** ELISA analysis for the concentration of IL-1β and IL-6 in the medium of adipocytes with treatment as indicated. **E** Oil red O staining assays of adipocytes with treatment as indicated. **F**, **G** Quantitative RT-PCR and Western blot analysis for mRNA and protein expression levels of TRIB3 and APN in adipocytes with treatment as indicated. **H** ELISA analysis for concentrations of IL-1β and IL-6 in the medium of adipocytes with treatment as indicated. **I** Oil red O staining assays of adipocytes with treatment as indicated. **J** Western blot analysis for protein expression levels of TRIB3, PPARγ, CEBPα and APN in adipocytes with treatment as indicated. **K** ELISA analysis for concentrations of IL-1β and IL-6 in the medium of adipocytes with treatment as indicated. **L** Western blot analysis for protein expression levels of SMAD2, p-SMAD2, SMAD3 and p-SMAD3 in adipocytes with treatment as indicated. **M** Western blot analysis for protein expression levels of TRIB3, SMAD3 and p- SMAD3 in adipocytes with treatment as indicated. **N** Oil red O staining assay of adipocytes with treatment as indicated. **O** Western blot analysis for protein expression levels of PPARγ, CEBPα and APN in adipocytes with treatment as indicated. Scale bar = 100 µm. Data were expressed as mean ± SEM of three independent experiments. Student’s *t* test was used for analysis of the data in **B**, **D**, **E**, **F**, **H**, **I**, **K**, **L**, **M** and **N**. *p < 0.05.

Previous studies have reported that several key cytokines secreted by cancer cells are involved in the transformation of adipocytes into CAAs [20, 21]. To investigate which cytokines are mainly involved in CAA formation, KEGG pathways were analyzed based on the top 200 DEGs between CAAs and control adipocytes identified using RNA-seq data (https://www.metascape.org). Our data showed that cytokine and cytokine receptor interaction and the TGF-β1 signaling pathway ranked in the top 10 (Fig. 4A), strongly suggesting the potential roles of TGF-β1 in CAA formation. Moreover, ELISA showed that the concentration of TGF-β1 was significantly higher in the culture medium of OC cells compared to the fresh medium (Supplementary Fig. 4A). Oil Red O staining assays demonstrated a concentration-dependent decrease in lipid droplet content in adipocytes incubated with TGF-β1 (Fig. 4B).

Furthermore, qRT-PCR and western blot analyses showed that TGF-β1 treatment significantly downregulated the mRNA and protein expression levels of PPARγ, CEBPα, and APN while upregulated TRIB3 expression in adipocytes (Fig. 4C, Supplementary Fig. 4B). In addition, TGF-β1 treatment significantly increased the contents of IL-1β and IL-6 in the culture medium of adipocytes (Fig. 4D). TRIB3 knockdown reversed TGF-β1-induced dedifferentiation of adipocytes (Fig. 4E–H), indicating that TGF-β1 induces CAA formation by upregulating TRIB3 expression.

It has been reported that TGF-β1 binds to membrane receptors to phosphorylate intracellular SMAD2 and SMAD3, which translocate into the nucleus to regulate target gene expression [22]. Given that TRIB3 has been identified as a target gene of TGF-β1/SMAD3 signaling [23], we therefore investigated whether the canonical TGF-β1/SMADs pathway contributes to TGF-β1-induced TRIB3 upregulation and formation of CAAs. As shown in Fig. 4I–K, the treatment of adipocytes with SD-208, a selective inhibitor of the TGF-β1 receptor, significantly attenuated the upregulation of TRIB3 and the formation of CAAs induced by TGF-β1. Western blot analysis showed that TGF-β1 treatment significantly increased the phosphorylation of SMAD3, but not SMAD2, in adipocytes (Fig. 4L), which was attenuated by treatment with SIS3 (a selective inhibitor of SMAD3) (Fig. 4M). In agreement with this, Oil red O staining and western blot analysis showed that SIS3 treatment also remarkably impaired TGF-β1-induced dedifferentiation of adipocytes (Fig. 4N, O). These data indicate that OC-derived TGF-β1 contributes to CAA formation by activating the SMAD3/TRIB3 signaling pathway.

### TGF-β1-induced CAAs promotes omental PMN formation through extracellular matrix remodeling

**Fig. 5:**
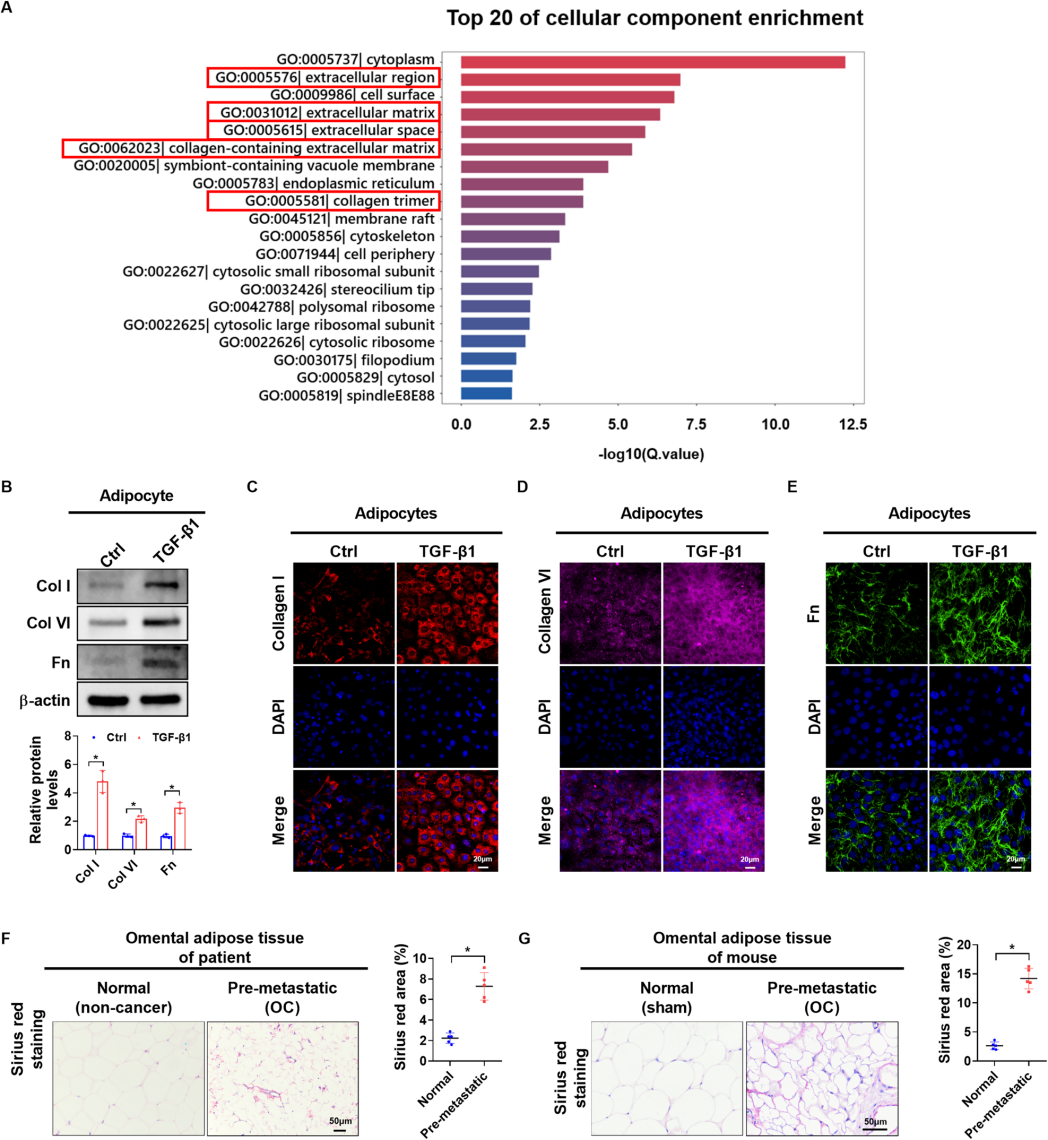
TGF-β1-induces CAAs promotes omental PMN formation through extracellular matrix remodeling. **A** Gene Ontology (GO) terms enriched by RNA-seq data from CAAs and control adipocytes. **B** Western blot analysis for protein expression levels in adipocytes with treatment as indicated (*n* = 3 independent experiments). **C**–**E** Representative immunofluorescence images of adipocytes with treatment as indicated (*n* = 3 independent experiments, Scale bar = 20 µm). **F** Sirius red staining images and collagen distribution in adipose tissues of patients (normal *n* = 5, pre-metastatic *n* = 6, Scale bar = 50 µm). **G** Representative Sirius red staining images and collagen distribution in adipose tissues of mice (*n* = 5 per group, Scale bar = 50 µm). Data were expressed as mean ± SEM. Student’s *t* test was used for analysis of the data in **B**, **F** and **G**. *p < 0.05.

Considering that the PMN is responsible for the metastatic tropism of cancer cells [2], we determined whether CAAs induce PMN formation in omental adipose tissues. RNA-seq data analysis showed that extracellular matrix-related components were significantly enriched in CAAs compared to control adipocytes (Fig. 5A). Previous studies have shown that CAAs overexpress collagen I (Col I), collagen VI (Col VI), and fibronectin (FN) to enhance metastasis by promoting cancer cell adhesion [11, 18, 24]. Similarly, western blotting and immunofluorescence staining assays showed that Col I, Col VI, and FN were also upregulated in adipocytes following TGF-β1 treatment (Fig. 5B–E). To validate these findings, we analyzed collagen distribution in omental adipose tissues of patients and orthotopic mice using Sirius red staining. Our results showed that pre-metastatic adipose tissues exhibited a significantly higher collagen distribution compared to control tissues (Fig. 5F–G). These findings indicate that TGF-β1-induced CAAs promote omental PMN formation through extracellular matrix remodeling.

### Inhibition of the TGF-β1 pathway prevents the formation of CAAs and PMN

**Fig. 6:**
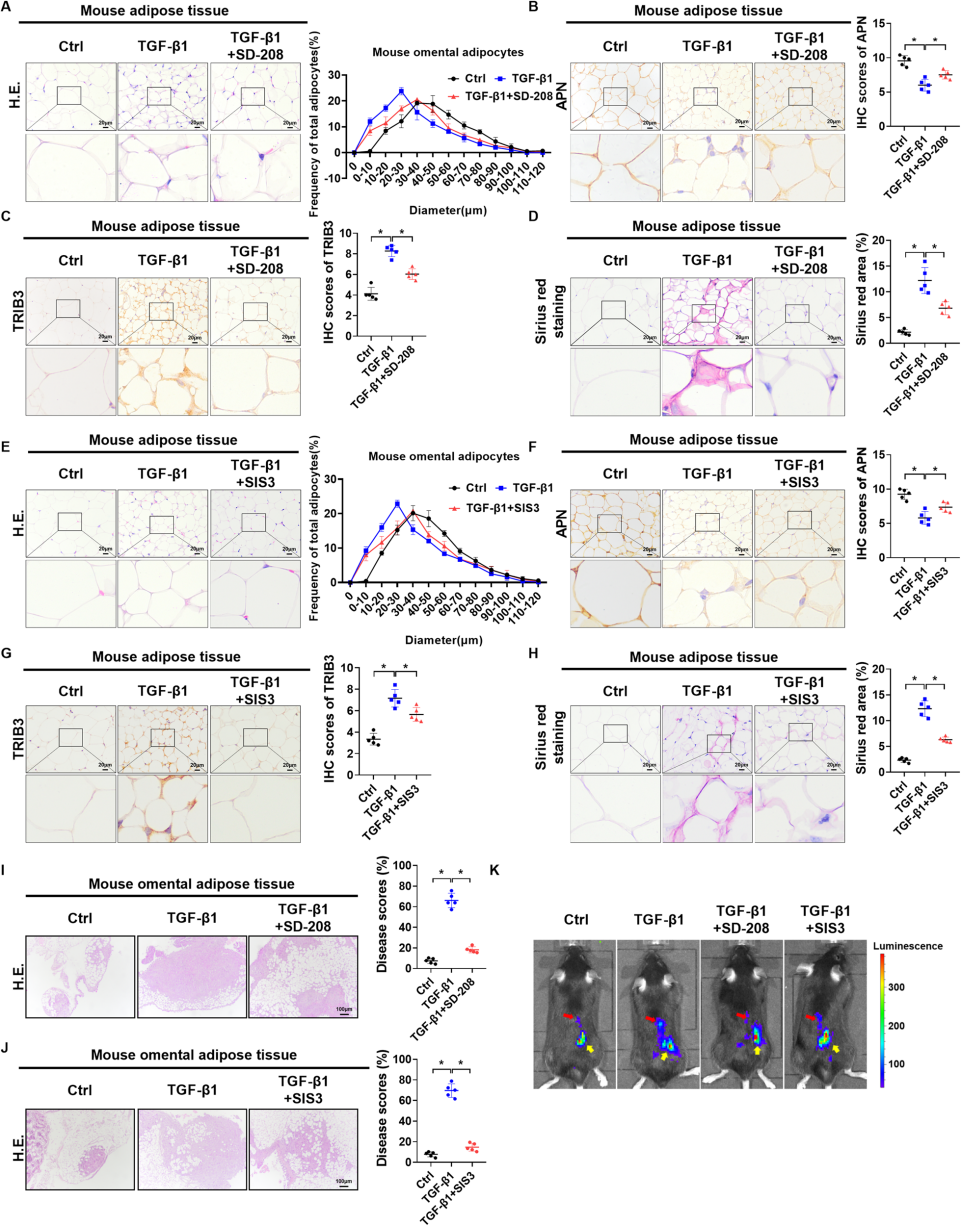
Inhibition of the TGF-β1 pathway prevents the formation of CAAs and PMN. **A** Representative H.E. images (left panel) and distribution of adipocyte diameter (µm) (right panel) in adipose tissues from mice with indicated treatments (*n* = 5 per group). **B**, **C** Representative IHC staining images and scores of APN and TRIB3 in adipose tissues from mice with indicated treatments (*n* = 5 per group). **D** Representative Sirius red staining images and distribution of collagen in adipose tissues from mice with indicated treatments (*n* = 5 per group). **E** Representative H.E. images (left panel) and distribution of adipocyte diameter (µm) (right panel) in mice adipose tissues (*n* = 5 per group). **F**, **G** Representative IHC staining images and scores of APN and TRIB3 in mice adipose tissues (*n* = 5 per group). **H** Representative Sirius red staining images in mice adipose tissues (*n* = 5 per group). **I**, **J** Representative H.E. images in omental metastases of OC cell (left). Scores of omental metastases were evaluated (right, *n* = 5 per group). **K** Orthotopic tumors in C57BL/6 mice were established by i.b. injection of ID8-Luc cells (10^6^) on day 0 (*n* = 5 per group). Different treatments were injected i.p. starting on day 7. On day 35, in vivo bioluminescence in mice were imaged. Representative images for each group are shown. Yellow and red arrows indicate primary OC and omental metastasis, respectively. Data were expressed as mean ± SEM. Student’s *t* test was used for analysis of the data in **B**–**D**, **F**–**J**. *p < 0.05.

To evaluate whether blocking the TGF-β1/SMAD3 signaling pathway inhibits CAAs and PMN formation, TGF-β1 was intraperitoneally injected into mice. The results of the H.E., IHC, and Sirius red staining assays showed that TGF-β1 treatment significantly reduced the diameter of omental adipocytes and the expression of APN (Fig. 6A, B) while increased TRIB3 expression (Fig. 6C) and collagen content (Fig. 6D). These effects of TGF-β1 on omental adipose tissues could be partly reversed by either TGF-β1 suppression with SD-208 treatment or SMAD3 suppression with SIS3 treatment (Fig. 6E–H). To evaluate the biological significance of the CAAs for the progression of OC cells, cell assays were performed after co-culture of adipocytes and ID8 cells. EdU assays showed that CAAs exerted limited effects on proliferation of OC cells (Supplementary Fig. 5A). However, wound healing and transwell assays (Supplementary Fig. 5B, C) indicated that CAAs significantly fortified the migration and invasion capacities of OC cells, which were attenuated by either SD-208 or SIS3 (Supplementary Fig. 5D, E). Moreover, omental metastatic burden analysis by H.E. staining indicated that TGF-β1 treatment significantly increased OC metastatic burden in the omentum, which could be attenuated by treatment with either SD-208 or SIS3 (Fig. 6I, J). In vivo bioluminescence in mice showed that the overall tumor burden in mice injected with ID8-Luc cells was significantly increased after TGF-β1 treatment, whereas the effects of TGF-β1 on omental metastasis could be partly reversed by SD-208 or SIS3 (Fig. 6K). In summary, our data indicate that blocking the TGF-β1 pathway prevents CAAs and PMN formation and reduces OC metastasis in vivo.

## Discussion

In the present study, we explored for the first time the functional roles of CAAs in OC omental tissues and found that CAAs contribute to PMN formation by remodeling the extracellular matrix to facilitate OC cell seeding. Mechanistically, OC-derived TGF-β1 functions in omental adipocytes and upregulates the expression of TRIB3 by phosphorylating SMAD3. Further, TRIB3 overexpression dedifferentiates mature adipocytes into CAAs by interacting with CEBPβ and inhibiting the CEBPβ-dependent induction of PPARγ and CEBPα. Compared to mature adipocytes, CAAs produce more collagen to induce omental PMN formation, facilitating the metastasis of OC cells. Furthermore, we found that blockage of the TGF-β1/SMAD3 signaling pathway by pharmacological inhibitors SD-208 and SIS3 prevents the induction of CAAs and PMN formation, consequently reducing OC metastatic burden.

White adipose tissue is now recognized to be dynamic, plastic and heterogenous, and is involved in a wide array of biological processes [25]. Previous studies have focused on CAAs around the primary cancer foci [10, 12, 13], however, the presence of CAAs in the target organs of metastasis remains unknown. In our study, the CAAs exist in the region surrounding omental metastases and PMN. We hypothesized that the adipocytes have already responded to cancer-derived TGF-β1 in a telecrine manner before OC cells arrival and established the PMN. When OC cells adhered to the omentum PMN and formed metastatic foci, CAAs distributed around the omental metastatic foci. Even the omental metastasis was formed, only sensitive adipocytes were responding to the OC cells-derived signal. We show for the first time that CAAs are present in premetastatic omental tissue before OC cell arrival. Furthermore, we found that TRIB3, an adipocyte dedifferentiation-related gene, plays a crucial role in CAA formation. TRIB3 is a pseudo-kinase containing a Ser/Thr protein kinase-like domain that lacks kinase activity [26, 27]. TRIB3 regulates kinase activity by competitively binding to its corresponding substrates. Several studies have proven that TRIB3 is involved in the metabolism of lipids and glucose by regulating Akt and other signaling pathways [27–30] and has been identified as a potent negative regulator of adipocyte differentiation [19, 31]. The suppression of preadipocyte differentiation is involved in a TRIB3-associated blockage of ERK signaling that prevents phosphorylation of regulatory sites of CEBPβ [19]. Additionally, TRIB3 exists in both the nucleus and the cytoplasm of preadipocytes and interacts with repression domain 1 of CEBPβ, inhibiting its ability to bind DNA and transactivate downstream adipogenic promoters [19]. Another study has demonstrated that TRIB3 physically interacts with PPARγ and suppresses its transcriptional activities to inhibit adipocyte differentiation in 3T3-L1 cells [31]. Consistent with these findings, we identified TRIB3 as a key regulator of the dedifferentiation of normal adipocytes into CAAs.

OC is a refractory disease with a high risk of metastasis to abdominal adipose tissue. Increasing evidence indicates that cancer-derived factors are responsible for the formation of CAAs [15]. For example, Wnt3a secreted by breast cancer cells induces adipocyte dedifferentiation by activating the Wnt/β-catenin signaling pathway in adipocytes [32]. Similarly, human MiaPaCa2 pancreatic cancer cells release Wnt5a, leading to adipocyte dedifferentiation [33]. Except for Wnt signaling, TNFA, and TGF-β1 have also been reported to induce the formation of CAAs by reducing the expression of PPARγ and CEBPα in adipocytes [15, 21, 34]. Although these studies revealed a link between cancer-derived factors and tumor-surrounding CAAs, the crosstalk between factors from primary cancer cells and CAAs in distant metastatic organs remains largely unclear. Our findings highlight TGF-β1 as a critical factor in promoting OC metastasis and potentially offer a new option for cancer treatment. The TGF-β1 signaling pathway governs key cellular processes under physiologic conditions but is deregulated in various pathologies, including cancer [22, 35–37]. The TGF-β1 pathway enhances cell proliferation, migration, invasion, and metastasis and inhibits immunosurveillance [35, 37]. In the canonical TGF-β1 signaling pathway, active TGF-β1 binds to the TGF-β1 receptor complex, a tetramer composed of two paired transmembrane serine/threonine protein kinases, to transact extracellular signals into cells successfully [22]. Activated TGF-β1 receptors initiate intracellular signaling by phosphorylating serine residues of SMAD2/3 to activate or repress the expression of target genes [22]. Future studies can investigate the potential of targeting other signaling pathways besides TGF-β1/SMAD3 in preventing CAA formation and OC metastasis.

We confirmed that OC cells secrete abundant TGF-β1, inducing the conversion of adipocytes to CAAs through activation of the classical signaling pathway, forming a PMN. Treatment with TGF-β1 receptor inhibitor SD-208 or SMAD3 phosphorylation inhibitor SIS3 effectively reduced TGF-β1-induced CAA formation. Consistent with our observations in OC metastasis, TGF-β1 has also been reported to upregulate TRIB3 expression in an SMAD3-dependent manner in fibroblasts in systemic sclerosis and murine alveolar type II epithelial cells [23, 38]. Based on extensive preclinical studies, pharmacological inhibition of the TGF-β1 pathway can be achieved using small molecule inhibitors, TGF-β1-directed chimeric monoclonal antibodies, ligand traps, antisense oligonucleotides, and vaccines, which have been evaluated in clinical trials [37]. Our study provides new evidence for TGF-β1 signaling as a therapeutic target for tumor metastasis. However, whether other signaling pathways involved in the OC-related CAA formation is still unclear and needs further investigation. Increasing evidence indicates that distant metastatic tissues are not passive recipients of circulating tumor cells and are selectively modified by the primary tumor before the arrival of tumor cells [2, 4]. These destined microenvironments are termed PMNs. Numerous studies have identified organ-specific PMNs in various types of tumors [2]. Consistent with this, we found that OC-induced CAAs remodel the extracellular matrix by secreting more type I collagen, type VI collagen, and fibronectin to facilitate metastatic OC cell transplantation. In line with our findings in OC, it has been demonstrated that breast cancer cells also act on adipocytes at the primary tumor site to promote the secretion of collagen I, VI, and fibronectin to remodel the extracellular matrix, thus enhancing cancer cell metastasis [11, 24]. Several clinical trials for targeting cellular and molecular components involved in the formation of PMN are performed [39]. Also, prospective preclinical or early clinical trials blocking TGF-β signaling pathway are developing [39].

In summary, we first demonstrated the formation and critical roles of CAAs in the omental metastatic tropism of OC and verified that targeting the TGF-β1/SMAD3 pathway is a new strategy to prevent OC metastasis. Moreover, the formation of CAAs and PMN in adipose tissues is conducive to the implantation of OC cells.

## materials and methods

### Reagents

Mouse TGF-β1 (Cat. No. HY-P73427), TGF-β1 receptor I kinase inhibitor SD-208 (Cat. No. HY-13227), and the SMAD3 inhibitor SIS3 (Cat. No. HY-13227) were purchased from MedChem Express, LLC (Newark, NJ, USA). The adipogenesis-associated reagent isobutylmethylxanthine (IBMX, Cat. No. I5879), and dexamethasone (DEX, Cat. No. D4902), rosiglitazone (Ros, Cat. No. R2408), and insulin (Cat. No. 91077C) were purchased from Sigma-Aldrich (St. Louis, MO, USA). The primary antibodies used in this study and their concentrations are listed in Supplementary Table S1.

### Cell culture

ES2, ID8, and 3T3-L1 cell lines were purchased from Beina Chuanglian Biotechnology Institute Co., Ltd. (Beijing, China) and the Chinese Academy of Sciences (Shanghai, China). Cells were cultured in RPMI‐1640 medium (Thermo Fisher Scientific, Inc., Waltham, MA, USA) or Dulbecco’s Modified Eagle Medium (Thermo Fisher Scientific, Inc., Waltham, MA, USA) supplemented with 10% fetal bovine serum (Gibco, Thermo Fisher Scientific, Inc., Waltham, MA, USA) and 1% penicillin/streptomycin solution, and incubated at 37 °C with 5% CO2. All cell lines were authenticated using short tandem repeat DNA testing for DNA Typing and tested mycoplasma negative.

### Differentiation of 3T3-L1 cells

3T3-L1 cells were cultured in induction medium (DMEM/F12 containing 10% FBS, 10 µg/mL insulin, 0.5 mM IBMX, 1 µM DEX, and 1 µM Ros) for 2 days. Subsequently, the induction medium was replaced with complete medium containing 10 µg/mL insulin. Mature adipocytes were identified by Oil Red O staining after eight days.

### Knockdown and overexpression of target genes

Lentiviruses for TRIB3 and CEBPβ were purchased from GeneChem (Shanghai, China) and used according to the manufacturer’s instructions. siRNAs were obtained from Tsingke Biotechnology Co., Ltd. (Beijing, China), and their sequences are listed in Supplementary Table S2. siRNAs were transfected using Lipofectamine 2000 reagent (Cat. No. 11668019; Invitrogen, Carlsbad, CA, USA) according to the manufacturer’s instructions.

### Conditioned media assays

OC cells were plated in duplicates in 6 well plates at a starting density of 2 ×10^5^ cells/well in 2 mL complete medium for 24 h to collect the conditioned media. The complete medium was then replaced with 2 mL of serum-free medium, and the cells were cultured for 24 h. The serum-free medium was collected and centrifuged to remove cell debris. Then the medium was filtered by a 0.22-µm filter to sterilize. For cell assays, conditioned medium was added to 6 well plates (3 mL/well) seeded with adipocytes for 48 h. For mice assays, conditioned media (300 µL) was i.p. injected into C57BL/6 mice daily for 21 days to induce the omental PMN formation [4].

### Oil red O staining

For Oil Red O staining, adipocytes were washed with PBS and fixed with 4% paraformaldehyde at 4 °C for 20 min. The adipocytes were then stained with 0.5% Oil Red O (O0625, Sigma-Aldrich) for 30 min to visualize the lipid droplets. Finally, the lipid droplets were photographed under a microscope (Olympus CX31), and the optical density at 540 nm was determined using a microplate reader (BioTek Synergy LX).

### Quantitative real-time PCR (qRT-PCR)

Total RNA was extracted from adipocytes using the Total RNA Kit I (OMEGA, R6834, USA) following the manufacturer’s instructions. Complementary DNA (cDNA) was synthesized using Transcriptor Reverse Transcriptase (RR036A; TaKaRa, Japan). Real-time PCR analysis was performed on a CFX 96 Real-Time System using an SYBR Green PCR kit (TaKaRa, RR820A, Japan). The *36B4* gene was used as an internal control. The primer sequences are listed in Supplementary Table S3.

### Western blot analysis

Total protein was extracted from lysed cells, separated by sodium dodecyl sulfate-polyacrylamide gel electrophoresis, and transferred onto PVDF membranes. The membranes were then incubated with primary antibodies overnight at 4 °C and HRP-conjugated second antibodies for 1 h at room temperature. β-actin served as an internal control. The blots were visualized using a chemiluminescence system (Pierce, USA). The antibodies used and their dilutions are listed in Supplementary Table S1.

### RNA-seq and data analysis

After extraction of total RNA from each sample using TRIzol reagent, the RNA samples were sent to LC-Bio Technology Co., Ltd. (Hangzhou, China) for RNA-seq analysis. The quantity and purity of each RNA sample were assessed using a NanoDrop ND-1000 (NanoDrop, Wilmington, DE, USA). cDNAs were generated using SuperScript II Reverse Transcriptase (Invitrogen, cat. 1896649, USA). Paired-end sequencing (PE150) was performed on an Illumina NovaSeq 6000 following the recommended protocol. Data analysis was conducted using Fastqc software (https://github.com/OpenGene/fastp). Standard differences in gene expression were selected with fold change >2 or <0.5, and a parametric F-test compared nested linear models (*P*-values < 0.05) using the R package edgeR.

### Immunofluorescence

Cells seeded on confocal dish were washed with PBS and fixed in 4% paraformaldehyde for 15 min at 4 °C, followed by 0.2% Triton-X permeabilizing for 10 min. The cells were then blocked in 2% bovine serum albumin (BSA) for 30 min and incubated with specific primary antibodies overnight at 4 °C. Subsequently, the samples were incubated with corresponding secondary antibodies for 1 h at 37 °C. DAPI was used at a dilution of 1:1000 for nuclear staining. Stained cells were observed using an Olympus FV3000 confocal microscope.

### Collection of human tissue samples

All human tissue samples were collected at the Xijing Hospital of the Fourth Military Medical University. Omental adipose tissues surrounding OC metastases (directly adjacent to the omental metastasis tumor within 1 cm of the edge of the omental OC metastases) [40] and matched adipose tissues distant from OC metastases (more than 2 cm from the edge of the omental OC metastases) [41] were obtained from 16 patients with high-grade serous ovarian cancer (HGSOC). In addition, omental adipose tissues were collected from five patients with benign tumors and six patients with Stage I/II HGSOC (without macroscopic omental metastases). Since OC is difficult to be detected early, we only collected omentum tissues from six patients with early-stage OC who underwent surgery at Xijing Hospital of the Fourth Military Medical University from March to August 2022. No statistical methods were used to predetermine the sample size.

### Establishment of the mouse model

Mice were given ad libitum access to food and water and maintained in a specific pathogen-free animal center under a 12 h light-dark cycle at room temperature (22 ± 0.5 °C). Mice were randomly assigned to each group (5 mice per group). An orthotopic OC mouse model was generated as previously described [8]. 6-week-old female C57BL/6 mice were anesthetized with 1% sodium pentobarbital, and depilatory cream was applied to remove hair from the backs of the mice to expose the operative site. A small incision was made in the dorsal flank using a retroperitoneal approach to expose the ovarian fat pad to the oviduct and ovaries. Mouse OC ID8 cells (10^6^ cells suspended in 10 µL Matrigel) were injected intrabursally (i.b.) into the ovary. Similar to previous reports [8], orthotopic tumors were palpable 3 weeks after i.b. injection of ID8 cells. Omental metastasis was confirmed by histological analysis at 5 weeks but was not detected at 4 weeks. Thus, pre-metastatic omental adipose tissues of mice (without macroscopic metastases) were obtained at 4 weeks following the i.b. injection of ID8 cells. Sham surgery was performed on mice by injecting 10 µL Matrigel i.b as control.

For pharmacological assays in vivo, mice in the TGF-β1 group were injected intraperitoneally (i.p.) with TGF-β1 (10 μg/g body weight) daily for 4 weeks, whereas the control group was injected with DMSO (1 μg/g body weight). In addition, SIS3 (10 μg/g body weight) or SD-208 (10 μg/g body weight) was injected i.p. 1 h before the TGF-β1 injection as TGF-β1 + SIS3 or TGF-β1 + SD-208 group, respectively (5 mice/group). Omentum tissues were collected after 4 weeks of treatment. For all animal studies, the tissue slides were independently assessed by two researchers blinded to the specific groups.

### Fluorescence imaging

For in vivo tracking, 10^6^ ID8 cells expressing Luciferase (ID8-Luc) were injected i.b. in C57BL/6 mice to established orthotopic tumors on day 0 (5 mice/group). Different treatments were injected i.p. starting on day 7 for 28 days. Mice were injected i.p. with D-luciferin (15 μg/g body weight, Bejing Jin Ming Biotechnology Co., Ltd.) and bioluminescence imaging was acquired by using IVIS Lumina II imaging system (Xenogen) to detect primary tumors and metastases on day 35. Representative images for each group are shown in Fig. 6K.

### Histological analyses

For hematoxylin–eosin (H.E.) staining, tissues were fixed in 4% formaldehyde for 24 h, embedded in paraffin, and sectioned at 3 µm thickness. Tissue sections were routinely stained with hematoxylin and eosin and imaged using an Olympus microscope CX31. The diameter of the adipocytes was measured using NIH ImageJ software (NIH, Bethesda, MD, USA) in at least three fields per slide. A statistical graph of the H.E.-stained sample architecture is presented in Fig. 6, highlighting the percentage of the area occupied by malignant cells. The disease score was quantified as the percentage of the tumor area in the whole tissue [42].

IHC was performed according to the manufacturer’s instructions using an IHC detection kit (Invitrogen, USA). Briefly, the tissue sections were deparaffinized and hydrated. The antigen was retrieved in a boiling citrate buffer. Subsequently, sections were blocked with 5% normal goat serum incubated with primary antibodies at 4 °C overnight. The dilution ratio of primary antibodies used was anti-adiponectin (1:800; Abcam, Cambridge, USA) and anti-TRIB3 (1:200; Novus Biologicals, USA). Next, the sections were incubated with the secondary antibody and 3,5-diaminobenzidine (DAB). The expression levels of the target proteins were independently assessed by two pathologists blinded to the patients’ clinical information according to the intensity and proportion of positively stained cells, as previously described [43].

### ELISA

To measure the levels of IL-1β and IL-6, omental adipose tissues were washed with ice-cooled PBS buffer. The dried tissues were then weighed and ground using a glass homogenizer. The ELISA was conducted following the manufacturer’s instructions. Briefly, different concentrations of standard substances and experimental samples were added to a microplate and incubated for 2 h at room temperature. An enzyme-labeled detection antibody was added to the wells. Subsequently, chromogenic substrate was added and incubated at room temperature for 20 min. The reaction was stopped by adding a stop solution to each well, and the absorbance was measured at 450 nm using a microplate reader (BioTek Synergy LX). Cytokine concentrations in OC cell media were determined using the same protocol.

### Sirius red staining

The adipose tissue samples were fixed in 4% formaldehyde for 24 h, embedded in paraffin, and sectioned at 3 µm. The sections were then routinely deparaffinized, hydrated, and incubated in Sirius Red solution for 1 h. Nuclei were stained with Mayer’s hematoxylin solution for 10 min. Subsequently, tissue sections were dehydrated and imaged under an Olympus microscope CX31.

### Statistical analysis

All data are presented as mean ± SEM from three independent experiments. Student’s *t* test, and two-way ANOVA followed by Bonferroni post-hoc tests were used under appropriate conditions. Two-tailed Student’ s *t* test was performed to examine differences between two groups. A *P*-value < 0.05 was considered significant. All statistical analyses were performed using SPSS (IBM version 17.0) and GraphPad Prism7 (GraphPad Software). No statistical methods were used to predetermine the sample size. Each sample size is indicated in the figure legend. No samples or animals were excluded from the analysis. Randomization was used in animal studies.

### Study approval

This study was approved by the ethics committee of the Fourth Military Medical University (approval number: KY20223036-1), and informed consent was obtained from all patients. All procedures were performed in compliance with relevant guidelines and regulations, including the principles outlined in the Declaration of Helsinki. All animal experiments were approved by the Animal Care Committee of the Fourth Military Medical University (approval number: 20220399). All procedures were conducted in accordance with relevant guidelines of welfare and ethical for laboratory animal of National Institutes for Food and Drug Control.

## Supplementary information

Ovarian cancer-derived TGF-β1 induces cancer-associated adipocytes formation by activating SMAD3/TRIB3 pathway to establish pre-metastatic niche

western blot

## Data Availability

The datasets generated during the current study are available from the corresponding author on reasonable request.
