# Supplementary material for: Ovarian cancer-derived TGF-β1 induces cancer-associated adipocytes formation by activating SMAD3/TRIB3 pathway to establish pre-metastatic niche
Source: Cell Death Dis. 2024 Dec 24;15(12):930. doi: 10.1038/s41419-024-07311-3 (PMC11668853; doi:10.1038/s41419-024-07311-3)
Supplement: Supplementary file 1 — Ovarian cancer-derived TGF-β1 induces cancer-associated adipocytes formation by activating SMAD3/TRIB3 pathway to establish pre-metastatic niche [file 41419_2024_7311_MOESM1_ESM.pdf]

Ovarian cancer-derived TGF-β1 induces cancer-associated adipocytes formation by activating SMAD3/TRIB3 pathway to establish pre-metastatic niche

Tian Gao[1, 2 #], Jibin Li[3#], Tianyi Cheng [2#], Xingguo Wang[2], Mengqing Wang[2], Zhiyang Xu[2], Yang Mu[2], Xianli He[1*], Jinliang Xing[3*], Shujuan Liu[2*]

[1]Department of General Surgery, Tangdu Hospital, Fourth Military Medical University, Xi'an, China, 710038.

[2]Department of Obstetrics and Gynaecology, Xijing Hospital, Fourth Military Medical University, Xi'an, China, 710032

[3]State Key Laboratory of Holistic Integrative Management of Gastrointestinal Cancers and Department of Physiology and Pathophysiology, Fourth Military Medical University, Xi'an, China, 710032

[#] These authors contributed equally to this work.

*Corresponding author.

Shujuan Liu, Department of Obstetrics and Gynecology, Xijing Hospital, Fourth Military Medical University, Xi'an, Shaanxi, China. Email: hanliu@fmmu.edu.cn

Jinliang Xing, State Key Laboratory of Holistic Integrative Management of Gastrointestinal Cancers and Department of Physiology and Pathophysiology, Fourth Military Medical University, Shaanxi, P. R. China. Email: xingjl@fmmu.edu.cn

Xianli He, Department of General Surgery, Tangdu Hospital, Fourth Military Medical University, Xi'an, Shaanxi, China. Email: wanghe@fmmu.edu.cn

RESULTS

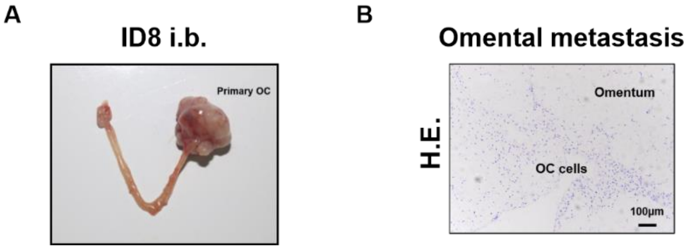

Fig. S1 The establishment of orthotopic OC mice models. (A) ID8 cells were injected i.b. into immunocompetent C57BL/6 mice, and primary OC was evaluated at 3 weeks thereafter. (B) Omental metastasis was observed by H.E. staining at 5 weeks after injection i.b. of .ID8 cells.

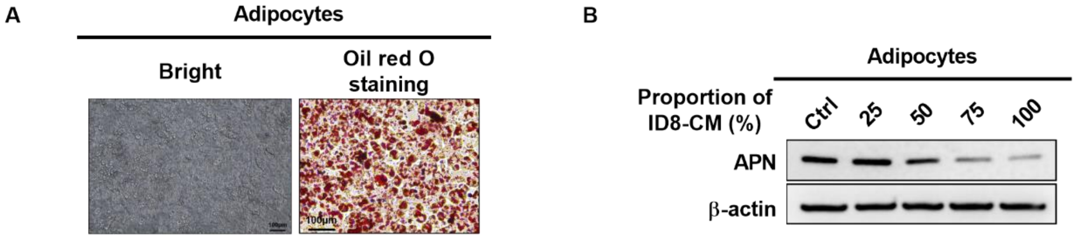

Fig. S2 Conditioned medium from OC cells dedifferentiated adipocytes into CAAs in vitro and in vivo. (A) Oil red O staining in 3T3-L1 cells-induced adipocytes. (B) The expression of APN was determined by western blot analysis in adipocytes treated with different concentrations of medium (Scale bar=100 μm).

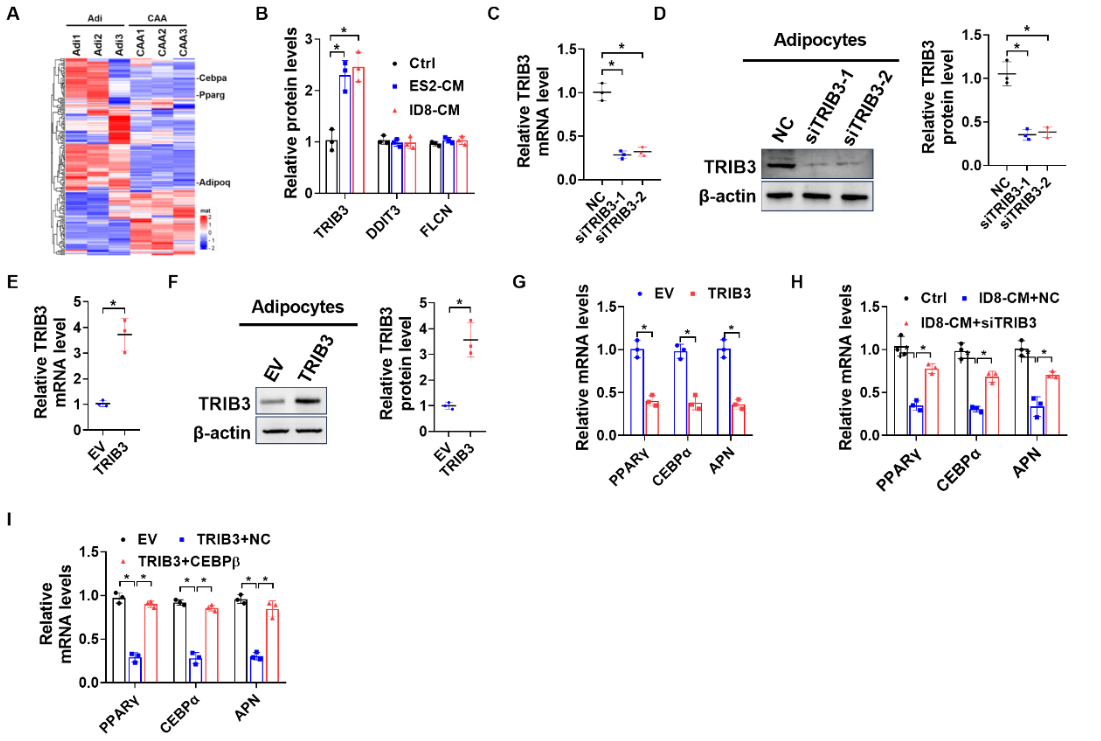

Fig. S3 Conditioned medium from OC cells induced CAAs formation via upregulating TRIB3 to suppress phosphorylation of CEBPβ. (A) Heatmap of differentially expressed genes (DEGs) between control adipocytes and CAAs. PPARγ, CEBPα, and APN were annotated. (B) Quantitative analysis of the blots in Fig 3B. (C-F) Quantitative real-time PCR and western blot analyses of TRIB3 expression in adipocytes with treatment as indicated. (G) Quantitative RT-PCR analysis for mRNA expression levels in adipocytes with treatment as indicated. (H) Quantitative RT-PCR analysis for mRNA expression levels in adipocytes with treatment as indicated. (I) Quantitative RT-PCR analysis for mRNA expression levels in adipocytes with treatment as indicated. Data were expressed as mean ± SEM of three independent experiments. Student's t-test was used for analysis of the

data. *$p < 0.05$.

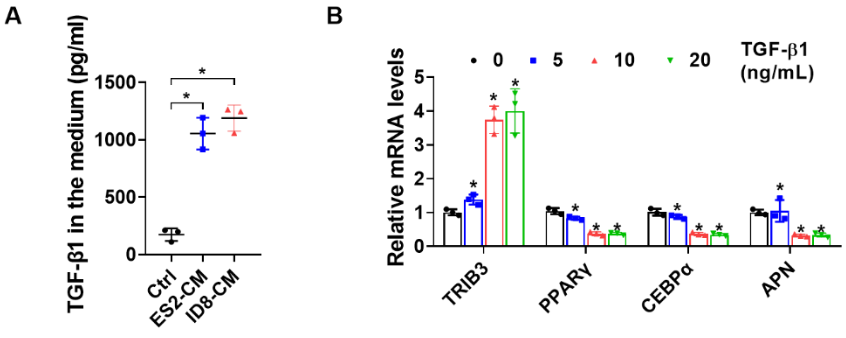

Fig. S4 OC-derived TGF-β1 contributed to CAAs formation by upregulating TRIB3 by activating SMAD3. (A) The concentration of TGF-β1 was measured by ELISA. (B) Quantitative RT-PCR analysis for mRNA expression levels of PPARγ, CEBPα, and APN in adipocytes with treatment as indicated. Data were expressed as mean ± SEM of three independent experiments. Student's t-test was used for analysis of the data. *$p < 0.05$.

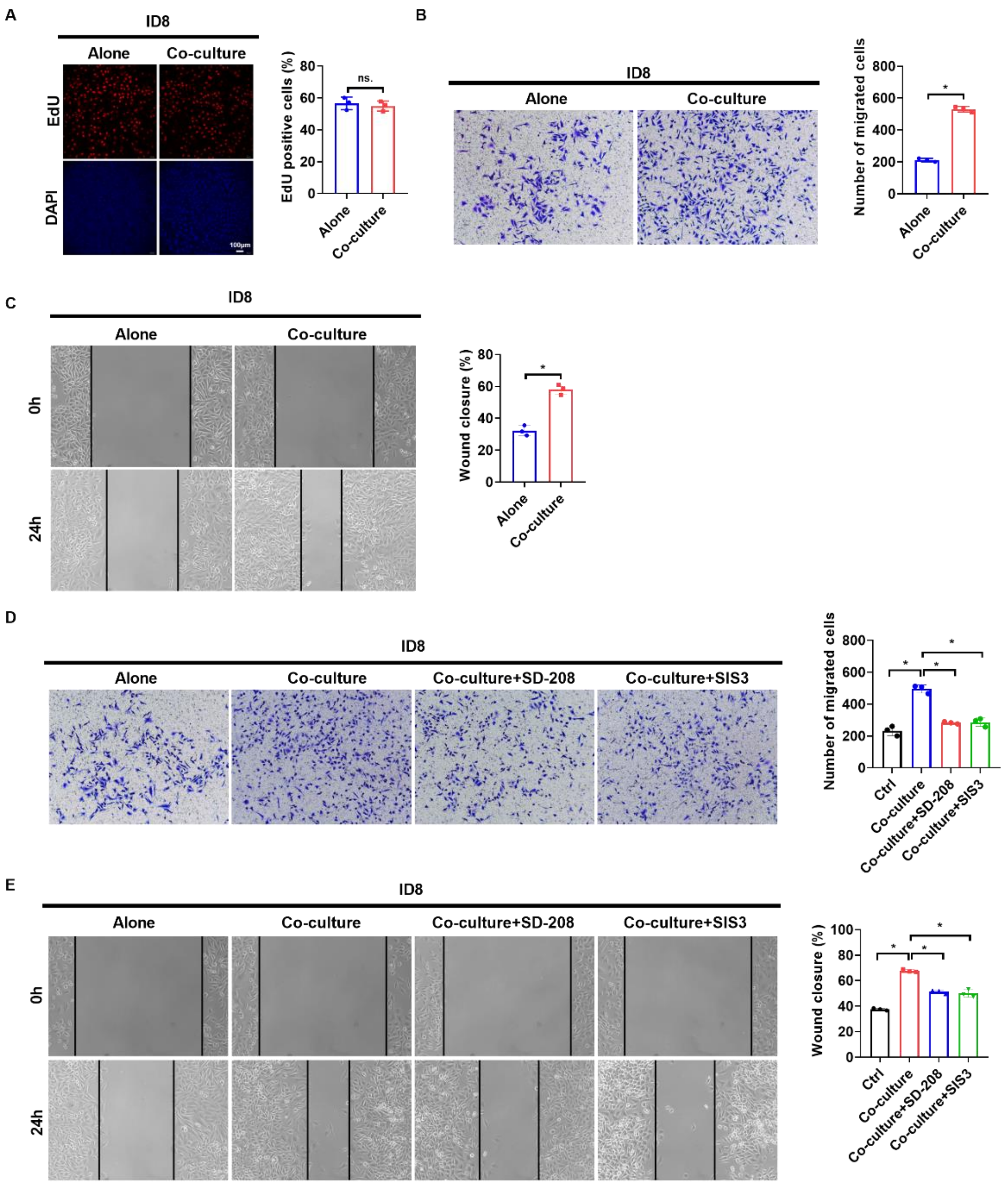

Fig. S5 **CAAs promote migration and invasion of OC cells.** ID8 cells were cultured with or without adipocytes, and then EdU (A), transwell (B) and wound healing (C) were performed for ID8 cells. Transwell (D) and wound healing assays (E) were conducted in ID8 cells cultured alone or with adipocytes or adipocytes treated with SD-208 or SIS3. Data are presented as the mean ± SEM of three independent experiments. Student's t-test was used for analysis of the data.. $^*p < 0.05$.

MATERIALS AND METHODS

EdU assay

To assess CAAs' effect on proliferation of OC cells, mature adipocytes and ID8 cells were co-cultured using a transwell co-culture system (6.5-mm inserts, Corning Costar, China). ID8 cells were seeded in the bottom layer of the transwell system, and adipocytes were seeded in the upper layer. Cancer cells cultured alone under similar conditions served as controls. A EdU-incorporation assay kit (Ribobio, Guangdong, China) was used following the manufacturer's protocol.

Transwell invasion assay

To assess CAAs' effect on invasion ability of OC cells, adipocytes and ID8 cells were co-cultured using a transwell co-culture system. ID8 cells were seeded in the upper layer, covered with Matrigel, of the transwell system, and adipocytes were seeded in the bottom layer. Cancer cells cultured alone under similar conditions served as controls. For pharmacological assays, the SD-208 or SIS3 was added in the medium of adipocytes in the bottom layer. After culture for 48 h, invaded cells were stained with crystal violet and counted by NIH ImageJ software. The average number of inserted cells in five randomly selected fields was calculated.

Wound healing assay

To assess CAAs' effect on migration of OC cells, mature adipocytes and ID8 cells were co-cultured using a transwell co-culture system (6.5-mm inserts, Corning Costar, China). ID8 cells were seeded in the bottom layer of the transwell system, and adipocytes were seeded in the upper layer. Cancer cells cultured alone under similar conditions served as controls. For pharmacological assays, the SD-208 or SIS3 was added in the medium of adipocytes. Wound healing assay was performed following the procedures as described previously (1).

Table S1 Primary antibodies used for Western blot, immunohistochemistry, and immunoprecipitation.

**Antibody**	**Company (Cat. No.)**	**Working concentration dilutions**
APN	Abcam (ab22554)	WB:1/1000; IHC:1/800
PPARγ	Abcam (ab45036)	WB:1/1000
CEBPα	Abcam (ab140479)	WB:1/1000
β-actin	ABclonal (AC026)	WB:1/3000
TRIB3	Novus (NBP2-82067)	WB:1/1000; IHC: 1/100; IP: 5 μg/μl
DDIT3	Abcam (ab11419)	WB:1/1000
FLCN	ABclonal (A14521)	WB:1/1000
CEBPβ	Cell Signaling (#3087)	WB:1/1000; IP: 5 μg/μl
p-CEBPβ	Cell Signaling (#3084)	WB:1/1000
SMAD2	ABclonal (A7699)	WB:1/1000
p-SMAD2	ABclonal (AP1342)	WB:1/1000
SMAD3	ABclonal (A19115)	WB:1/1000
p-SMAD3	ABclonal (AP0727)	WB:1/1000
Col I	Proteintech (14695-1-AP)	WB:1/1000; IF:1/200
Col VI	Proteintech (17023-1-AP)	WB:1/1000; IF:1/200
FN	ABclonal (A12977)	WB:1/1000; IF:1/50

Table S2. Sequences of siRNA

	Sense (5'-3')
siCtrl	GCGCGCUUUGUAGGAUUCG
siTRIB3-1	CGAGUGAGAGAUGAGCCUG
siTRIB3-2	CAGAAGAATGGTACAAATCCAAG

Table S3. Primers used in qPCR analysis and sequences of siRNA

**Gene**	**Forward Primer**	**Reverse Primer**
H-36b4	TCGTGGAAGTGACATCGTCTTT	CTGTCTTCCCTGGGCATCA
H-APN	GCAGAGATGGCACCCCTG	GGTTTCACCGATGTCTCCCTTA
H-IL-1β	TCAGCCAATCTTCATTGCTCAA	TGGCGAGCTCAGGTACTTCTG
H-IL-6	AGGGCTCTTCGGCAAATGTA	GAAGGAATGCCCATTAACAACAA
M-APN	TGGAATGACAGGAGCTGAAGG	TATAAGCGGCTTCTCCAGGCT
M-IL-6	GCCCACCAAGAACGATAGTCA	CAAGAAGGCAACTGGATGGAA
M-IL-1β	ACCATGGCACATTCTGTTCAAA	GCCCATCAGAGGCAAGGA
M-36b4	GCAGACAACGTGGGCTCCAAGCAGAT	GGTCCTCCTTGGTGAACACGAAGCCC
M-TRIB3	CTTTTGGAACGAGAGCAAGG	GTGTTGTGGGTATCTGAAGG
M-PPARγ	CCCTGGCAAAGCATTTGTAT	GAAACTGGCACCCTTGAAAA
M-CEBPα	CAAGAACAGCAACGAGTACCG	GTCACTGGTCAACTCCAGCAC
M-TRIB3	CTTTTGGAACGAGAGCAAGG	GTGTTGTGGGTATCTGAAGG

References

1. Li J, Huang Q, Long X, Zhang J, Huang X, Aa J, Yang H, Chen Z, and Xing J. CD147 reprograms fatty acid metabolism in hepatocellular carcinoma cells through Akt/mTOR/SREBP1c and P38/PPARalpha pathways. *J Hepatol.* 2015;63(6):1378-89.
