# Supplementary material for: Ovarian cancer-derived TGF-β1 induces cancer-associated adipocytes formation by activating SMAD3/TRIB3 pathway to establish pre-metastatic niche
Source: Cell Death Dis. 2024 Dec 24;15(12):930. doi: 10.1038/s41419-024-07311-3 (PMC11668853; doi:10.1038/s41419-024-07311-3)

Figure 2C

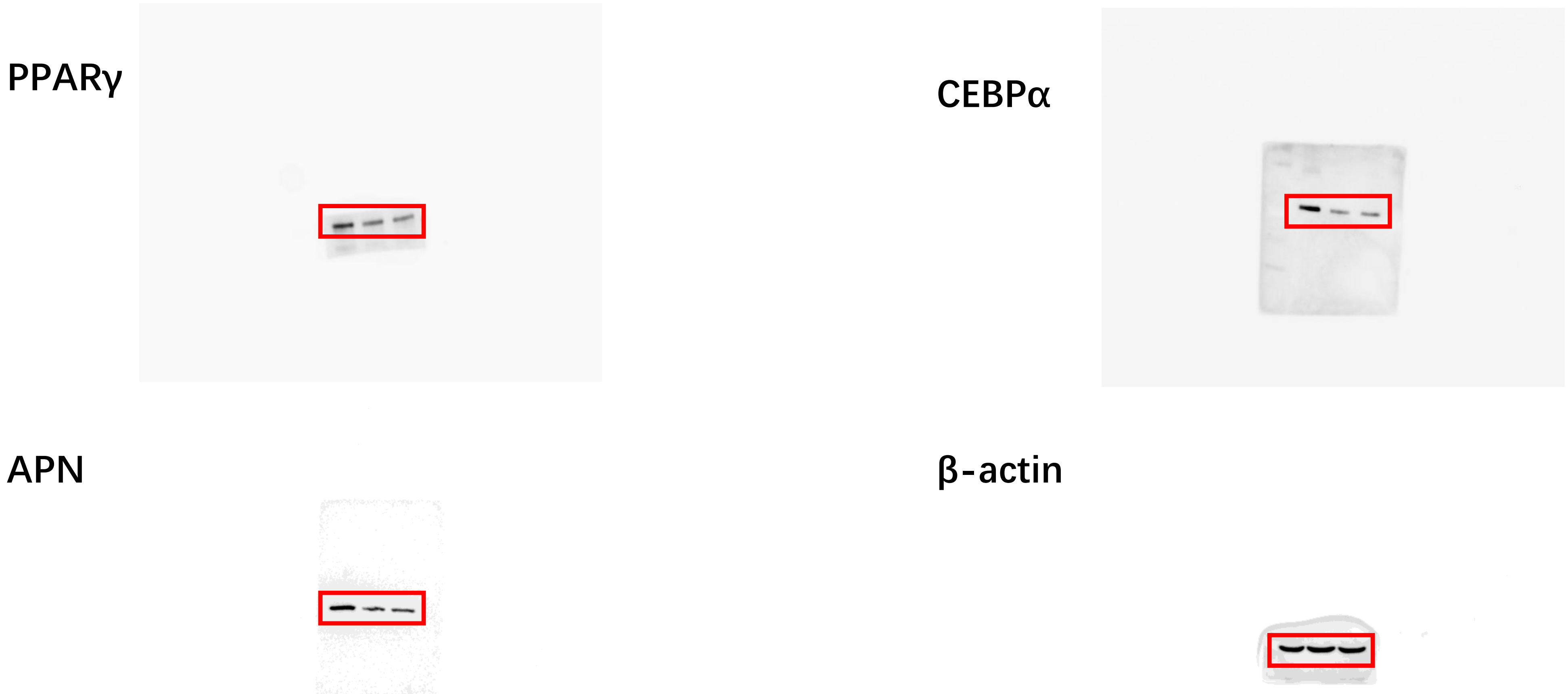

Figure 3B

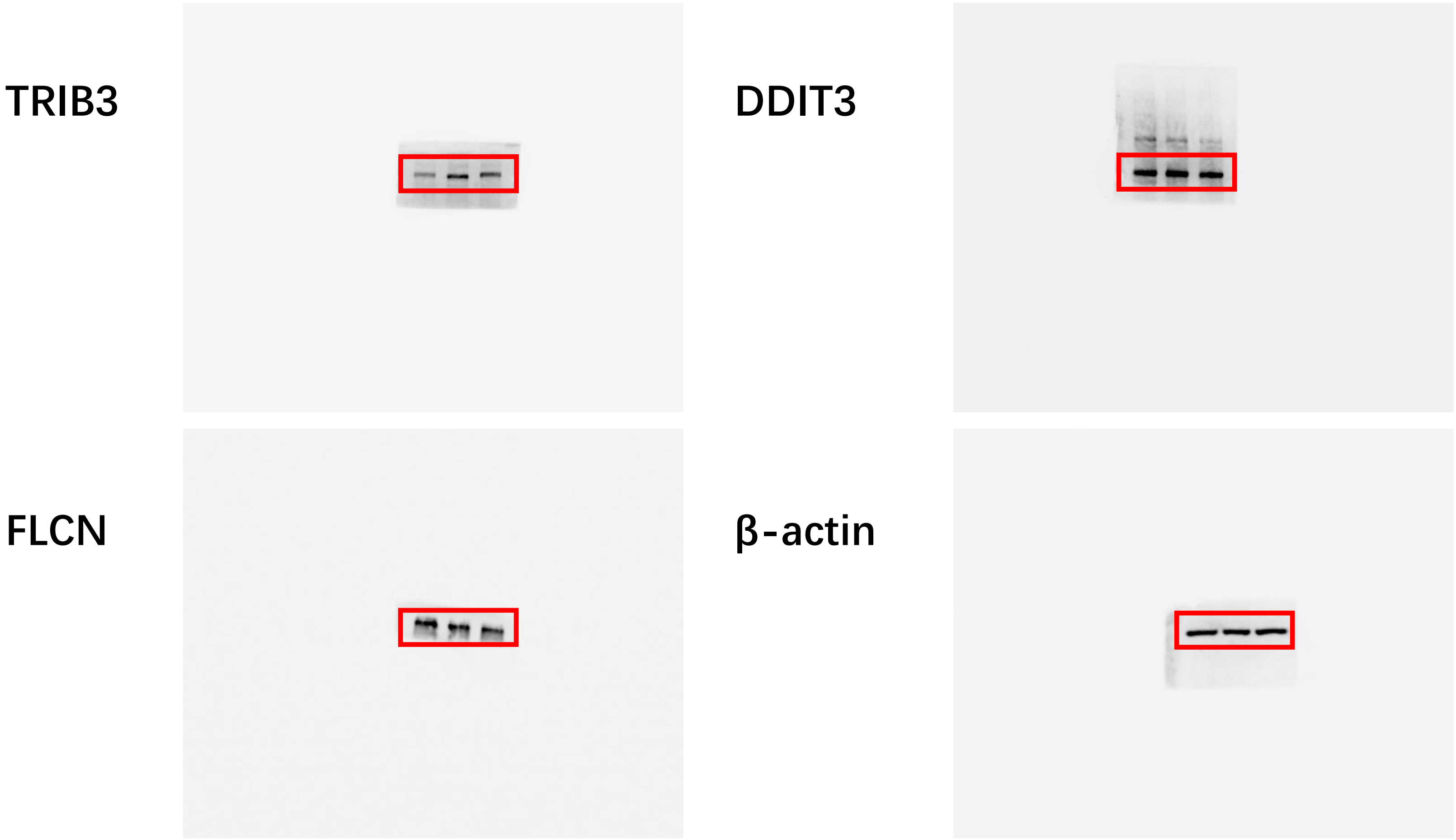

Figure 3D

PPARγ

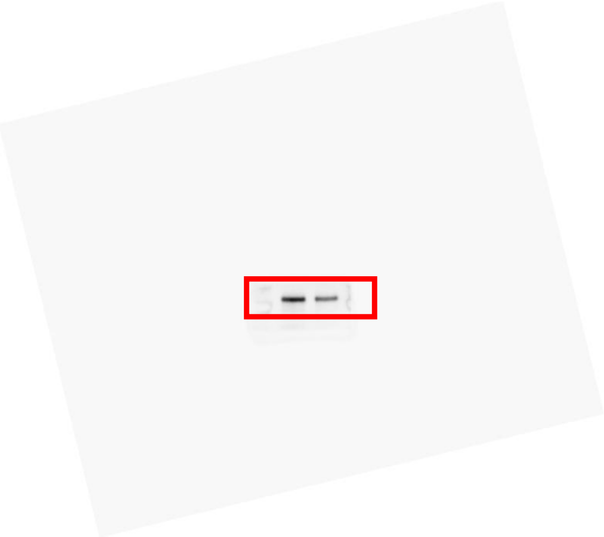

CEBPα

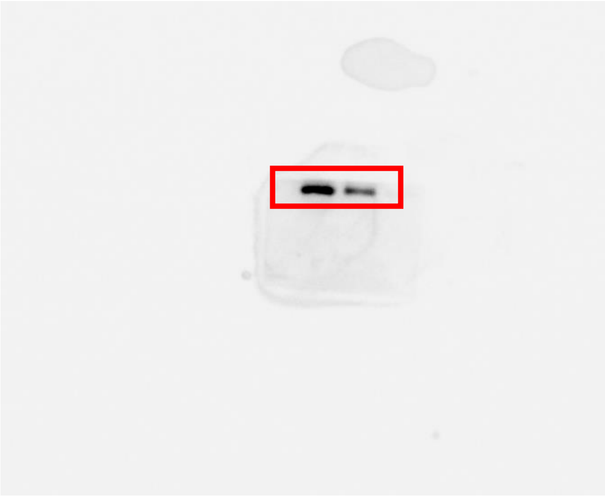

APN

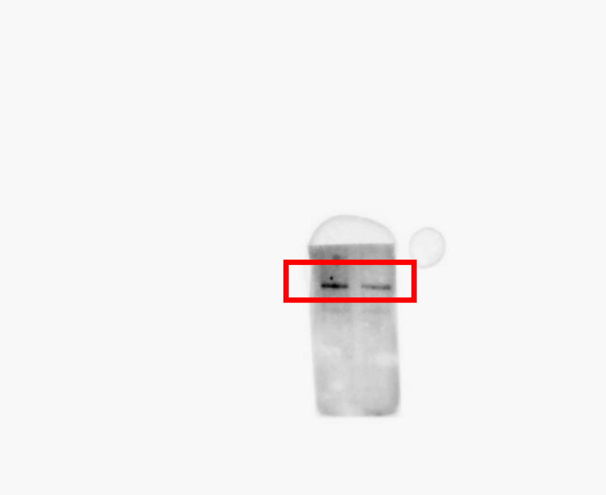

β-actin

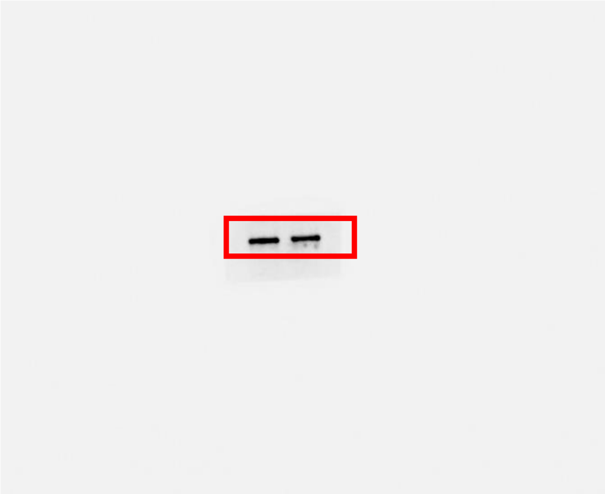

Figure 3G

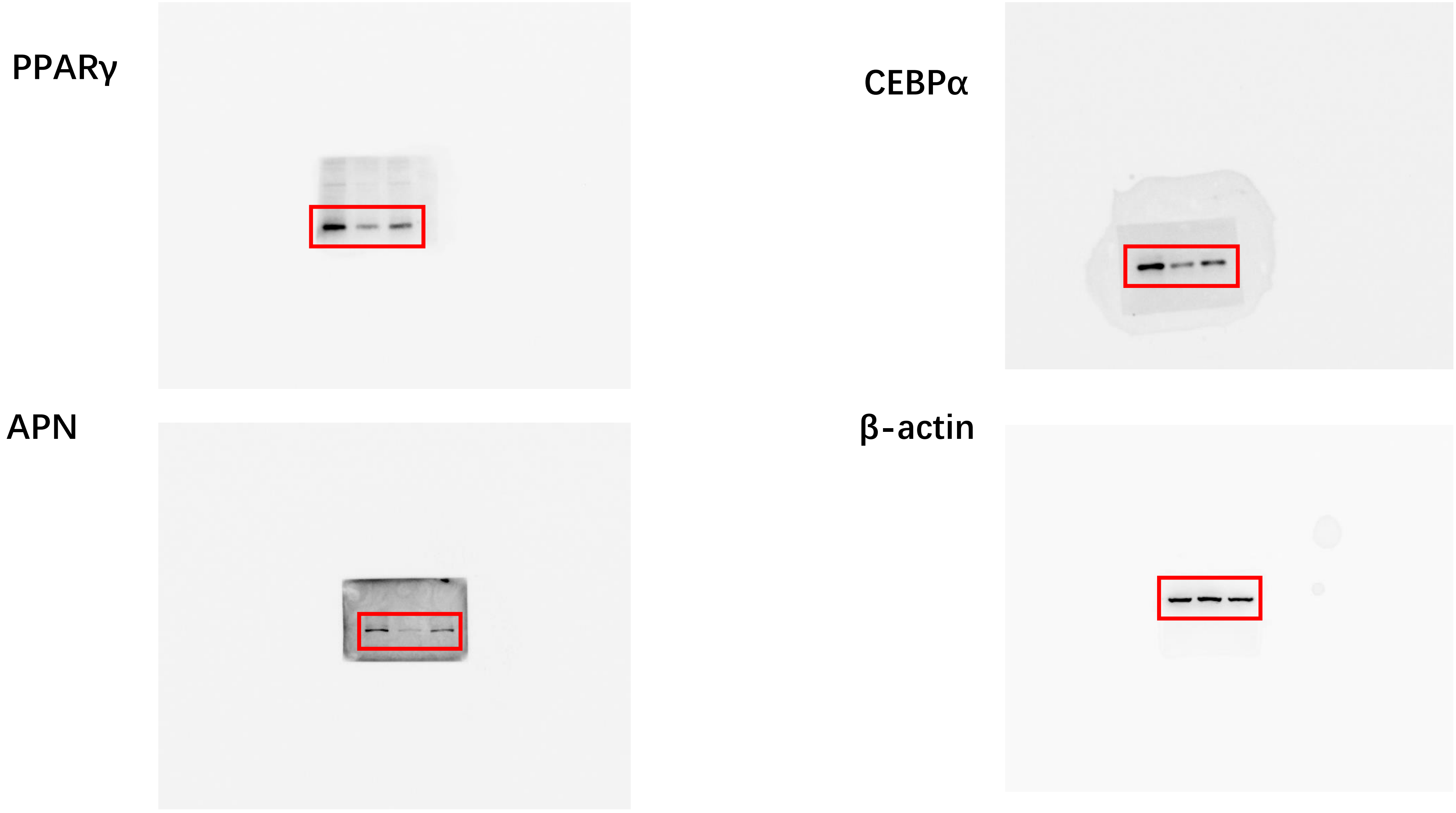

Figure 3I

TRIB3

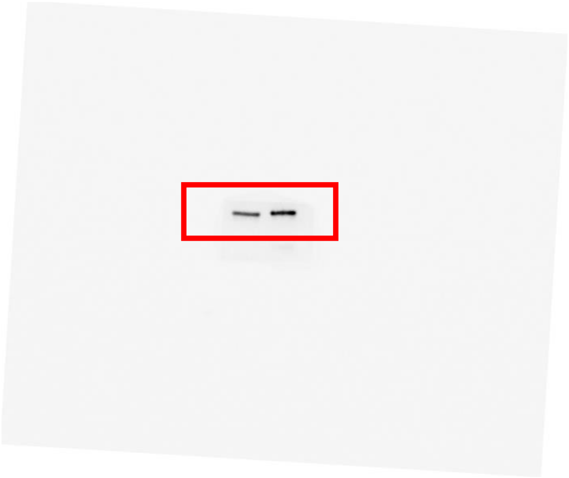

CEBPβ

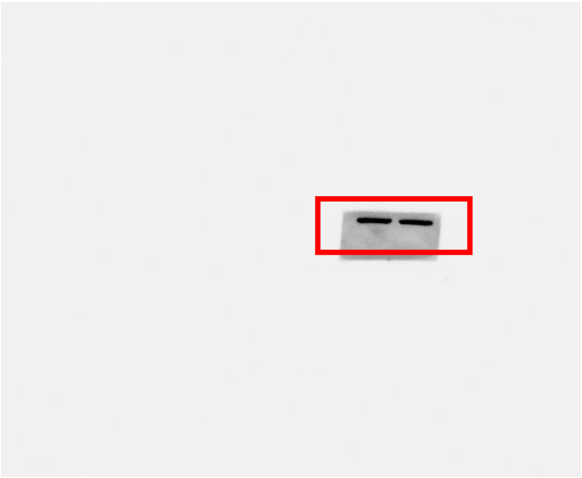

p-CEBPβ

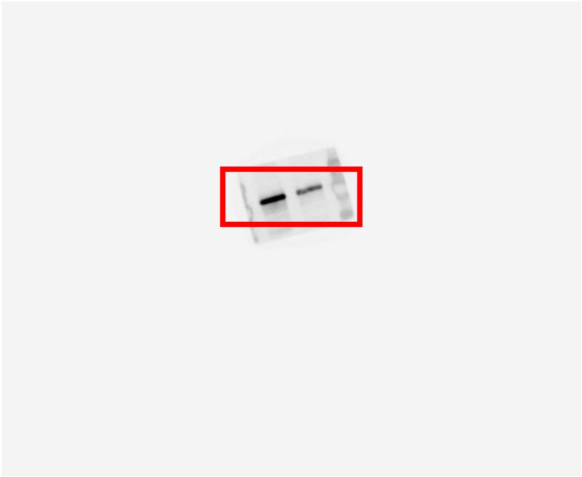

β-actin

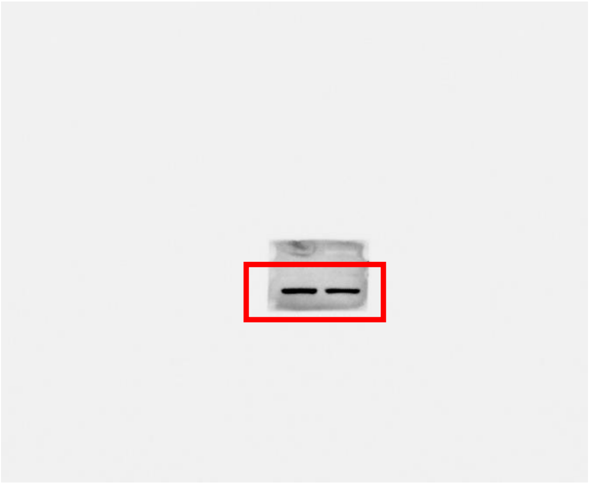

Figure 3J

CEBPβ

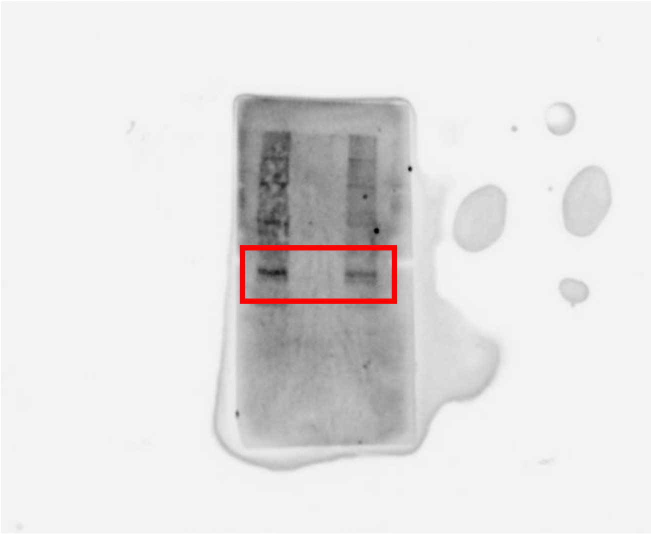

TRIB3

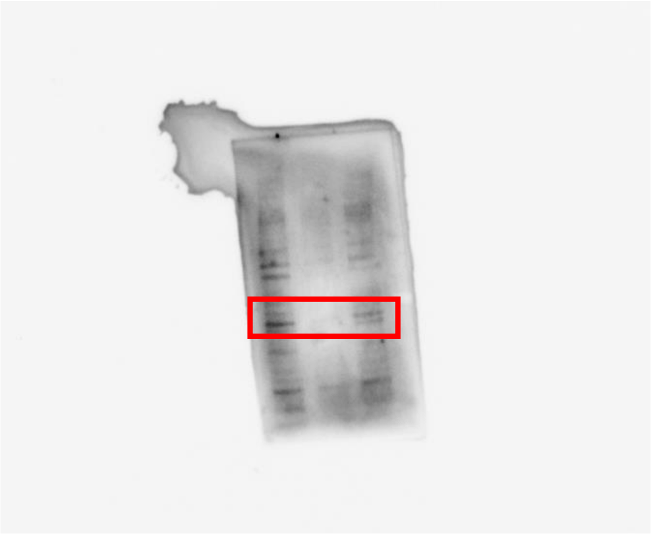

TRIB3

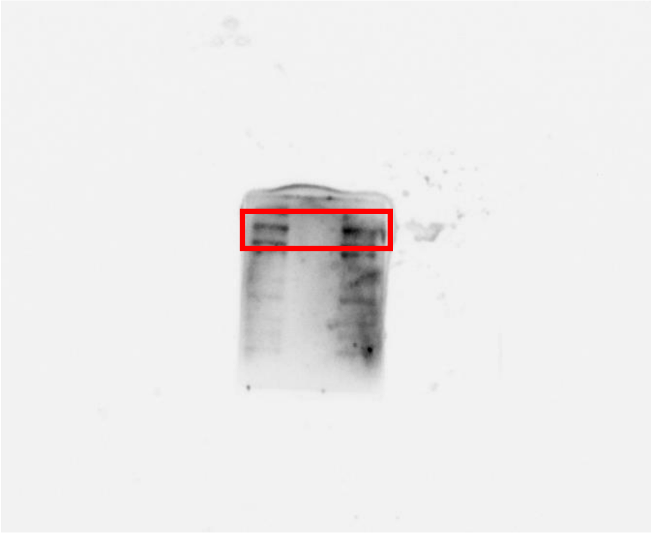

CEBPβ

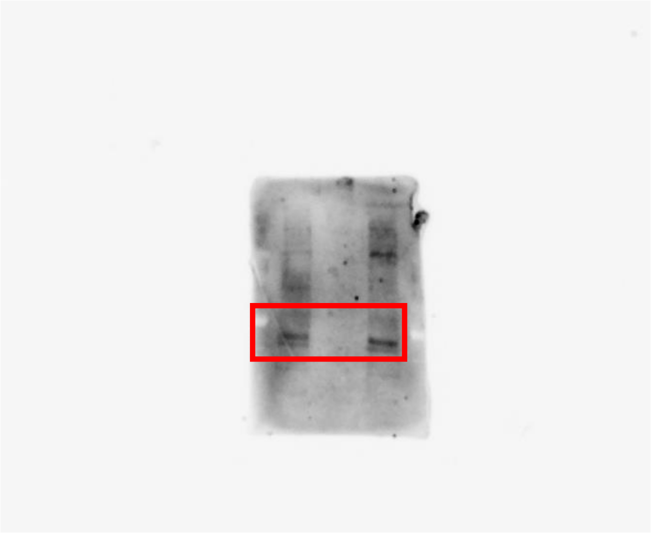

Figure 3L

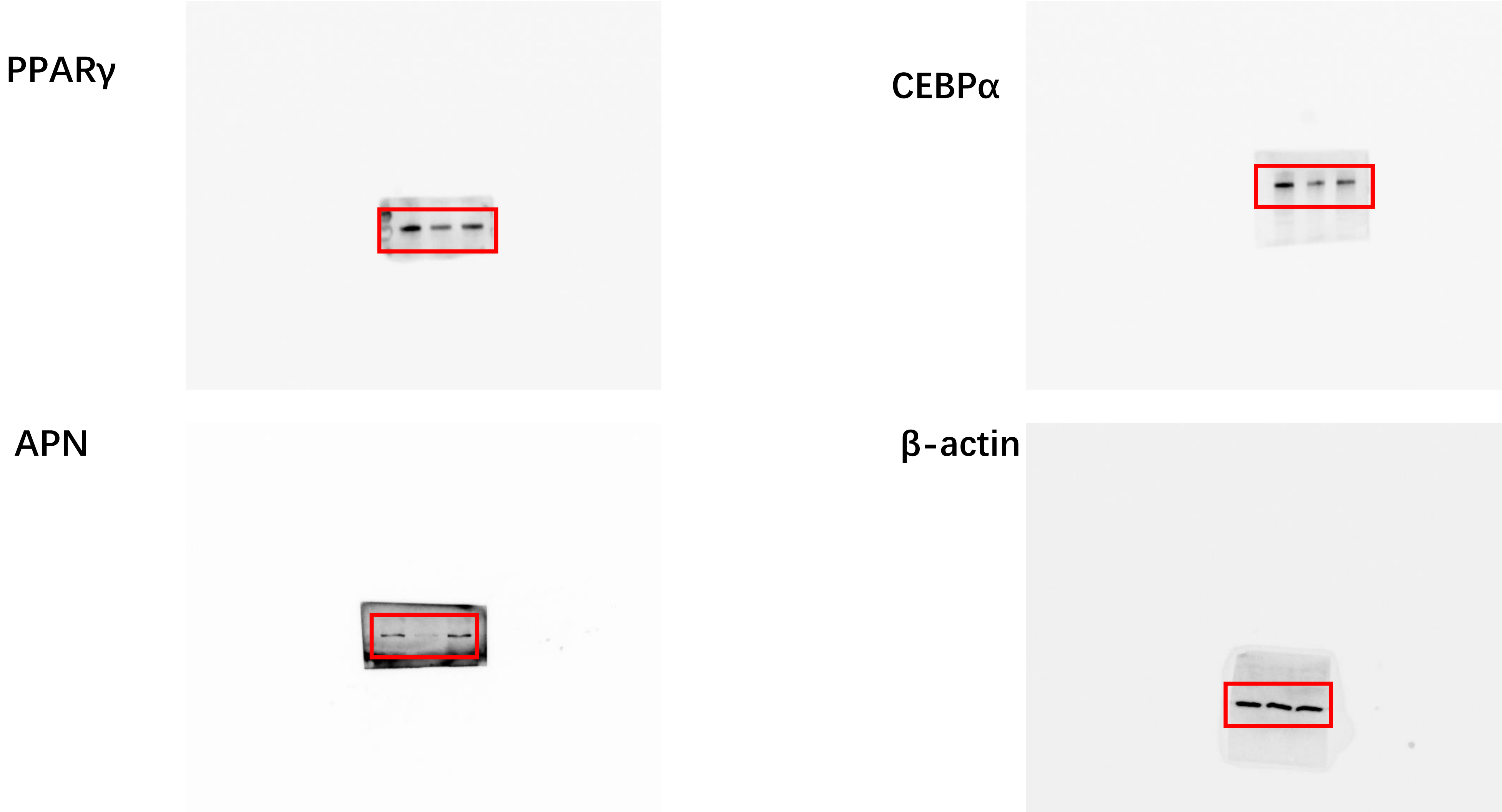

Figure 4C

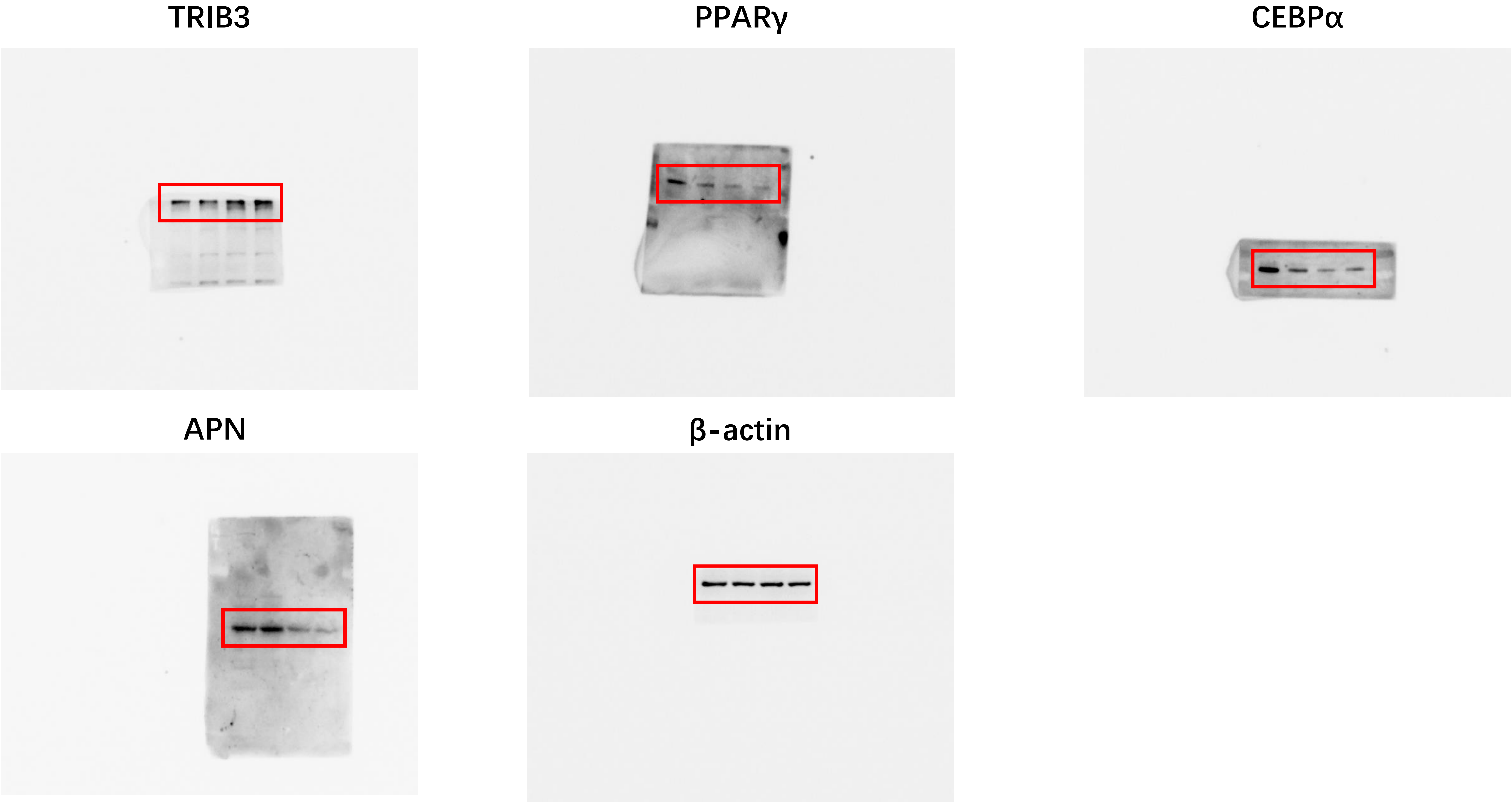

Figure 4G

TRIB3

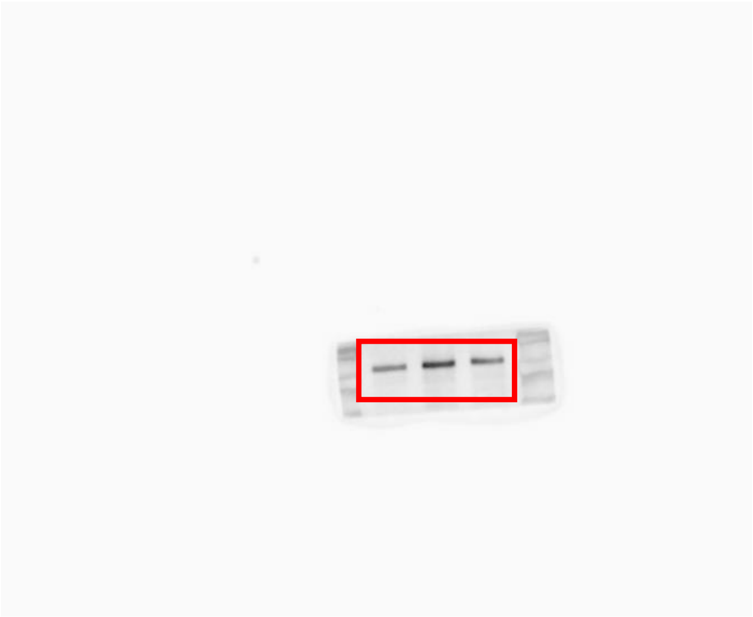

APN

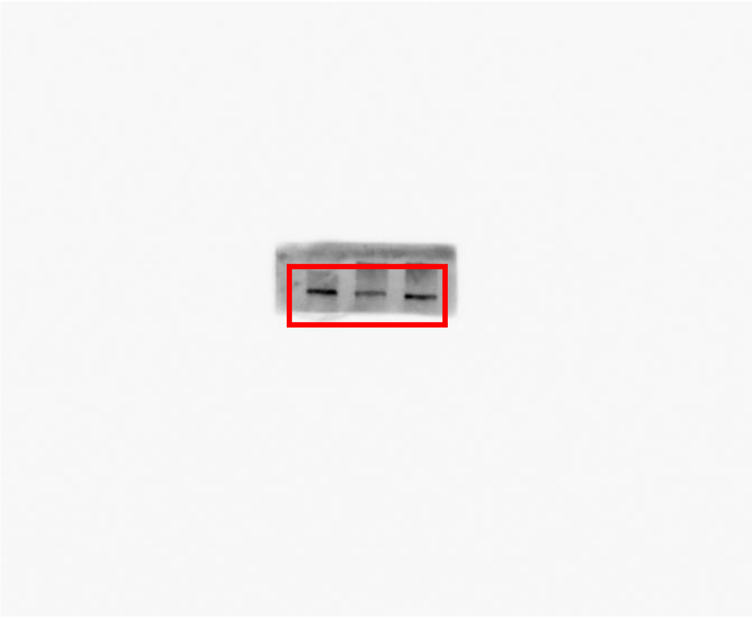

β-actin

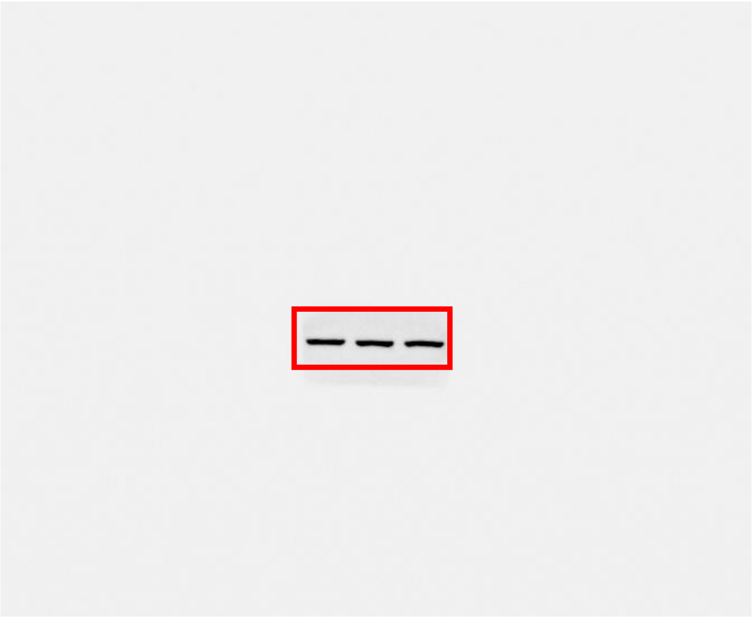

Figure 4J

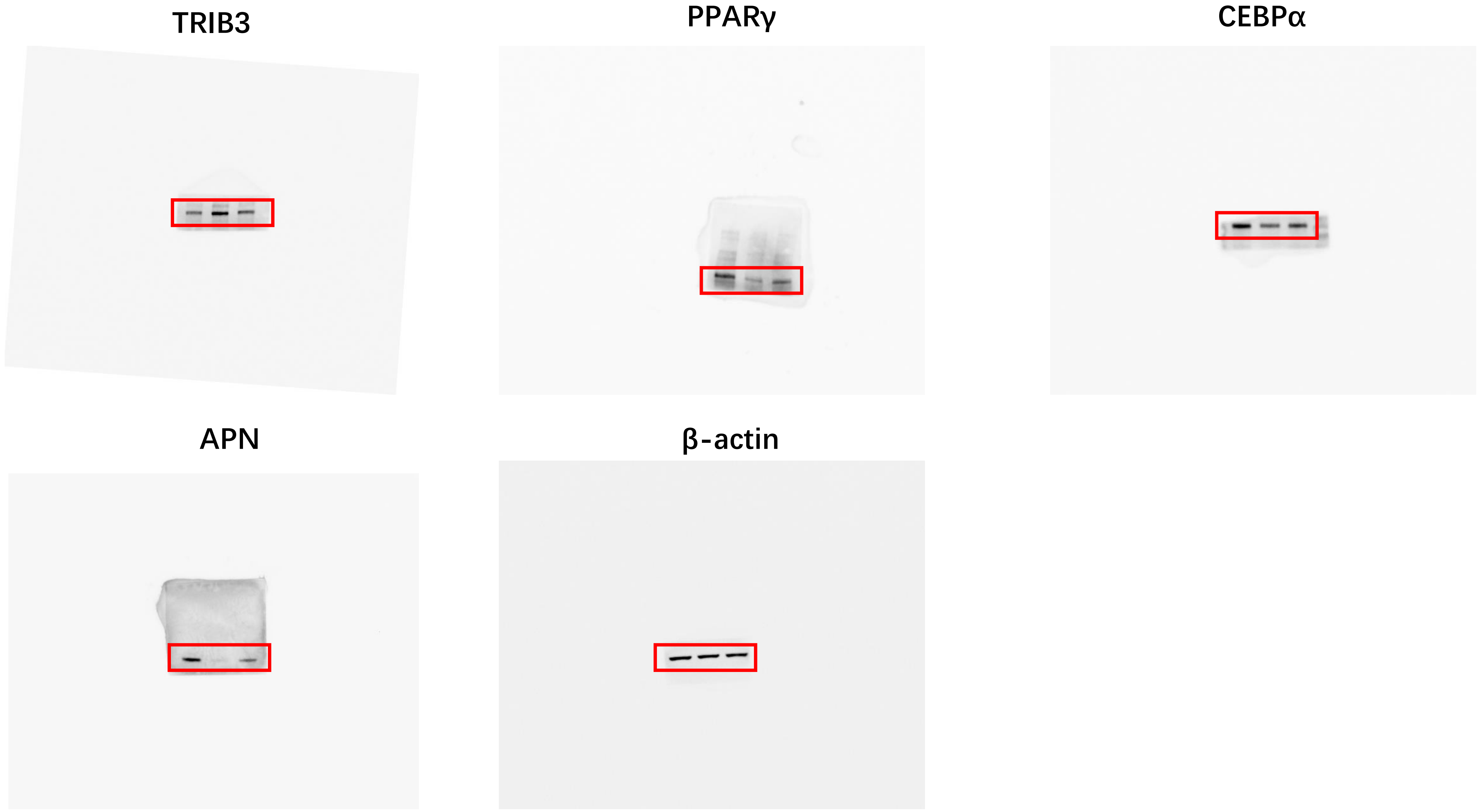

Figure 4L

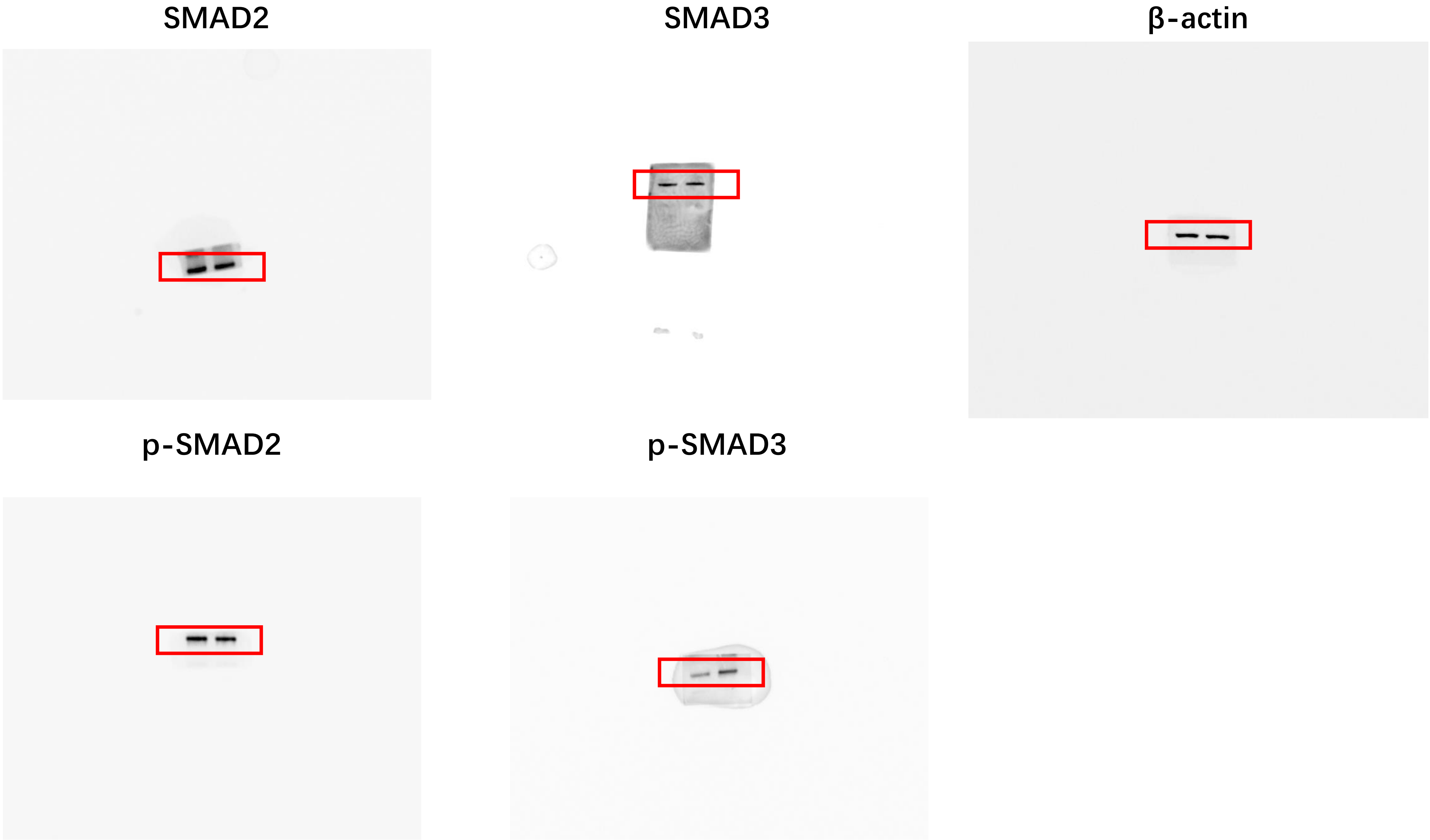

Figure 4M

TRIB3

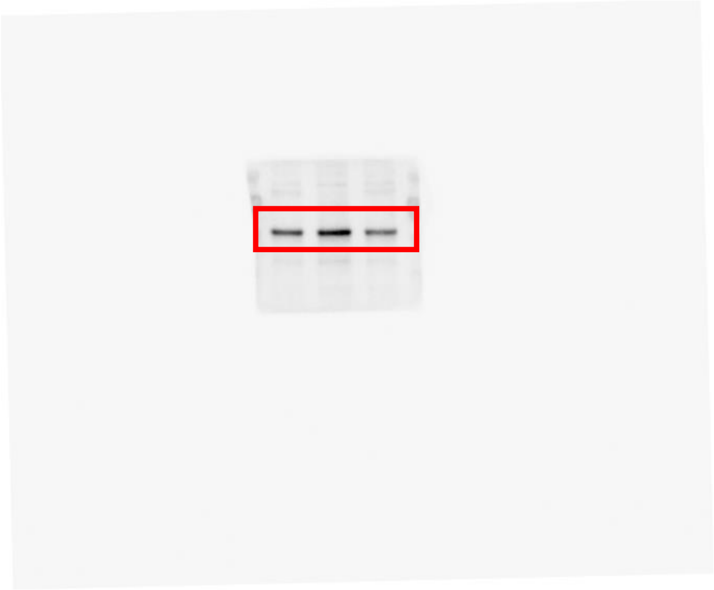

SMAD3

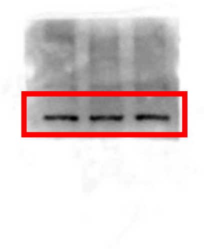

p-SMAD3

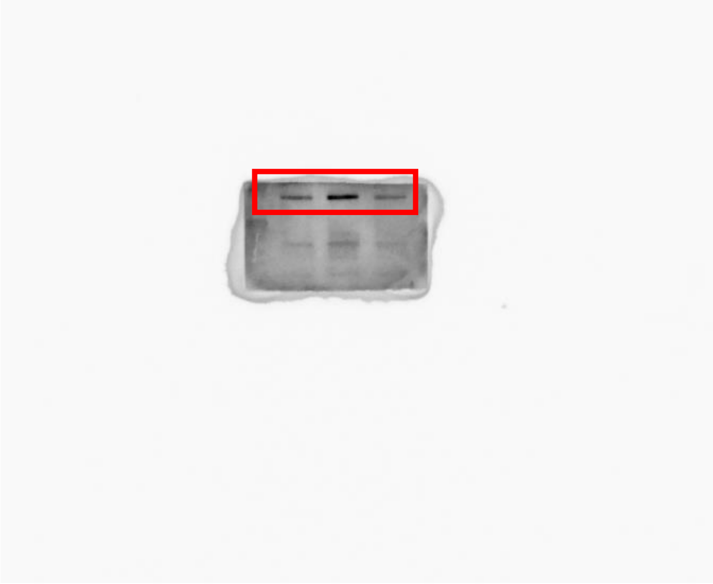

β-actin

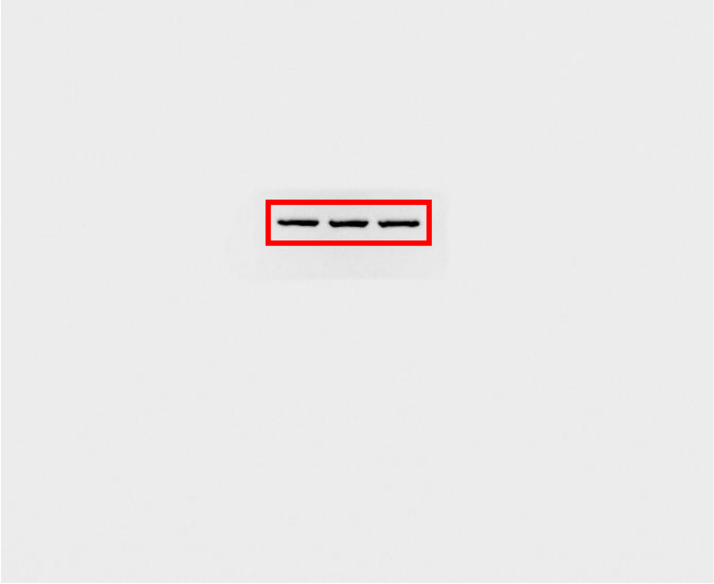

Figure 4O

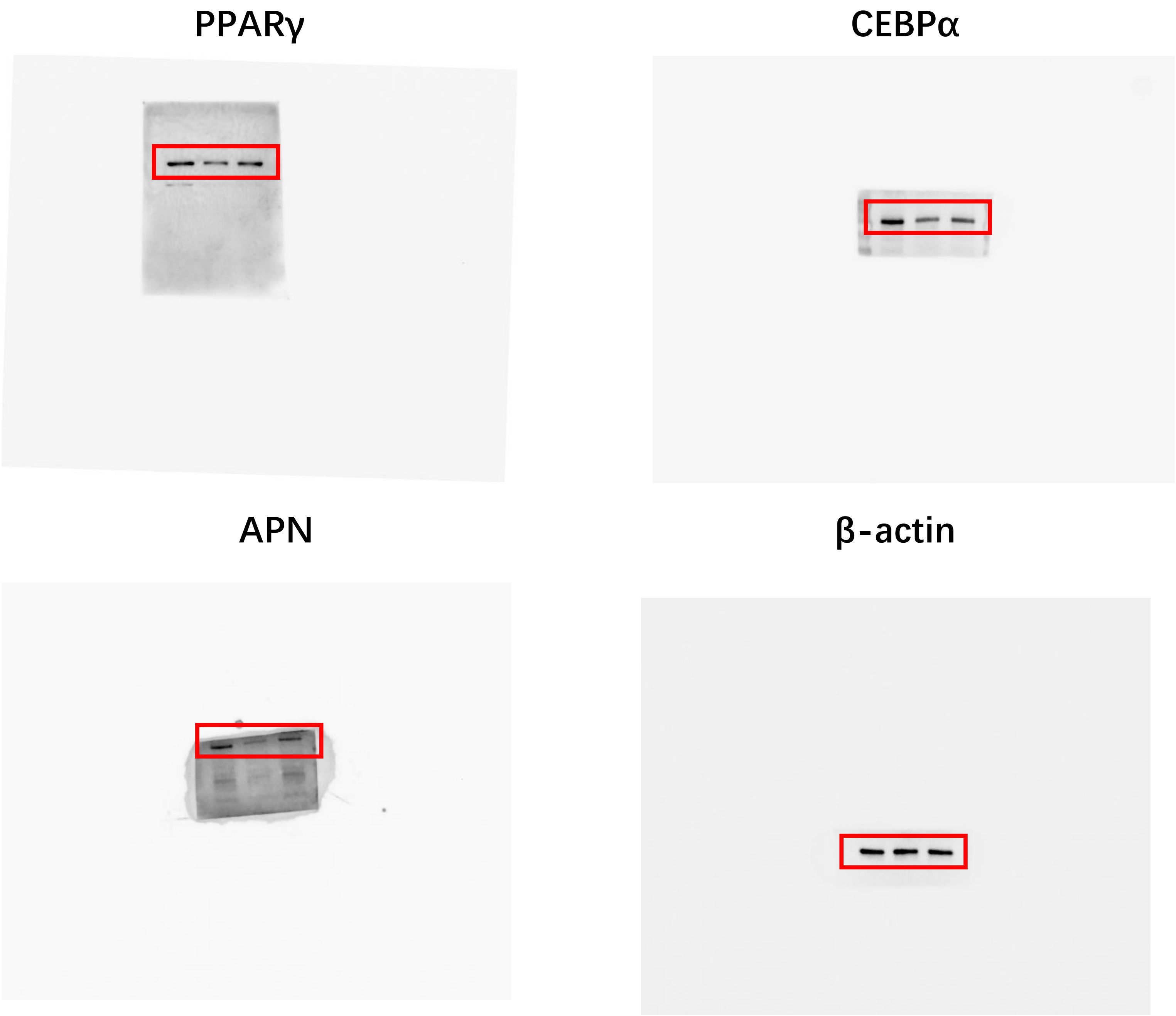

Figure 5B

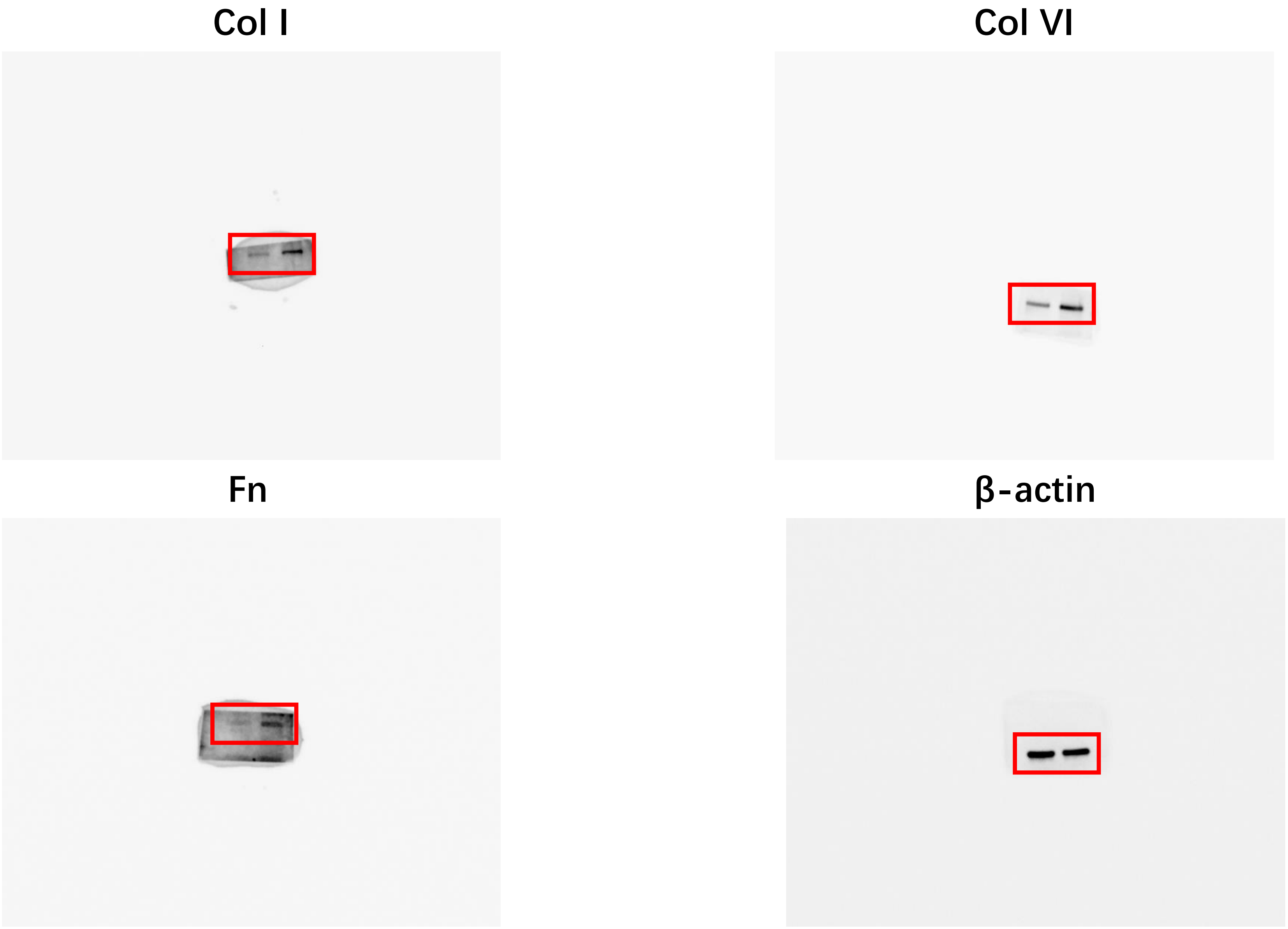

Figure S2B

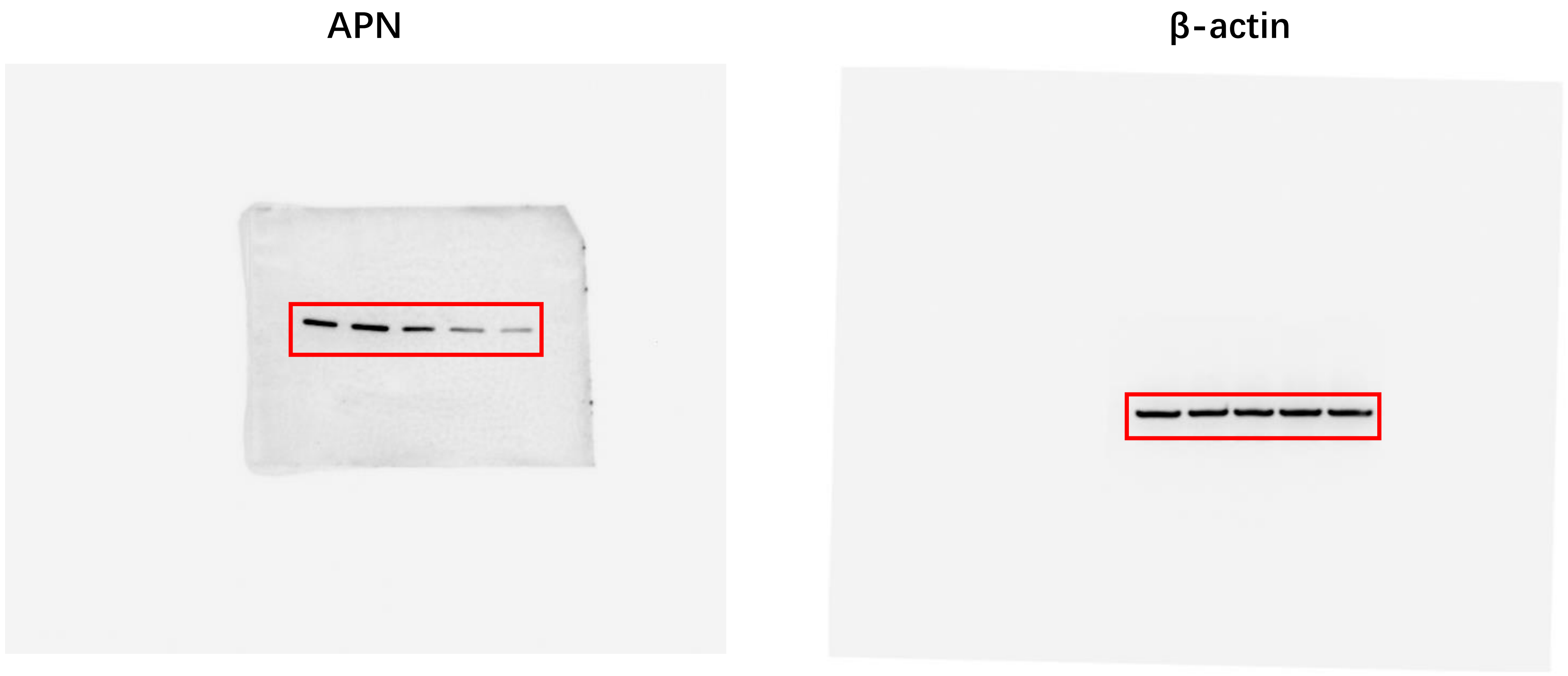

Figure S3D

TRIB3

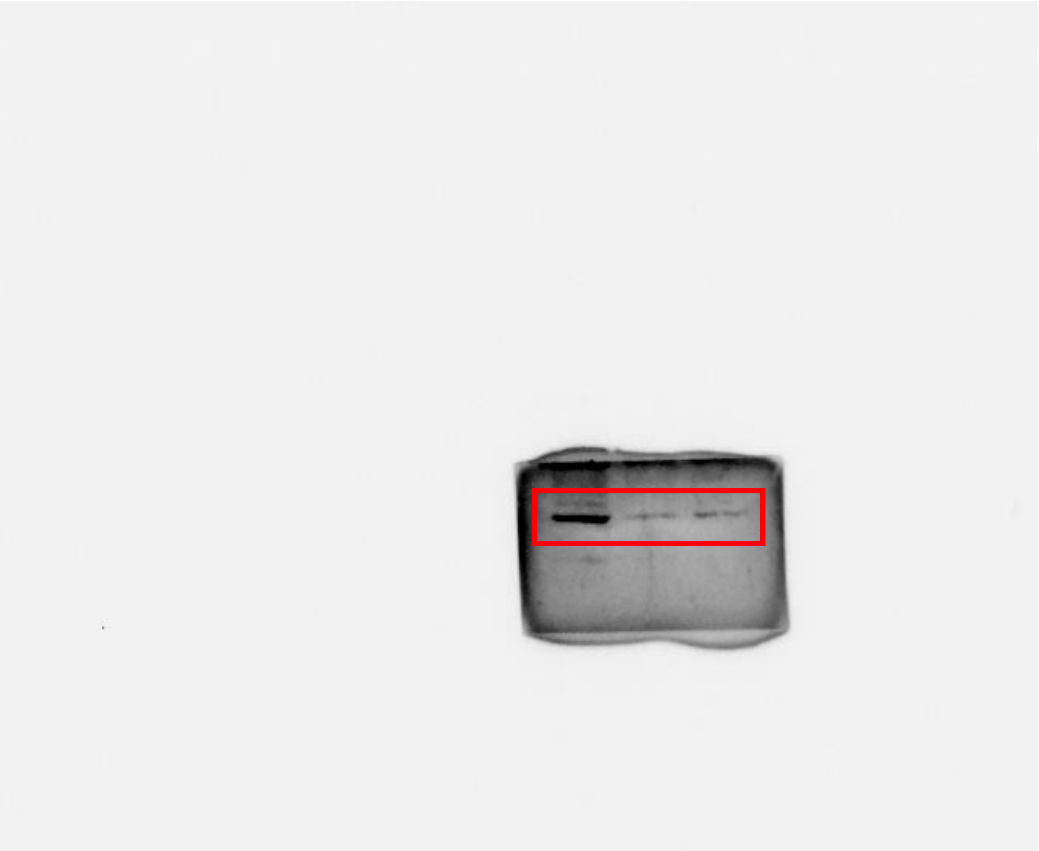

β-actin

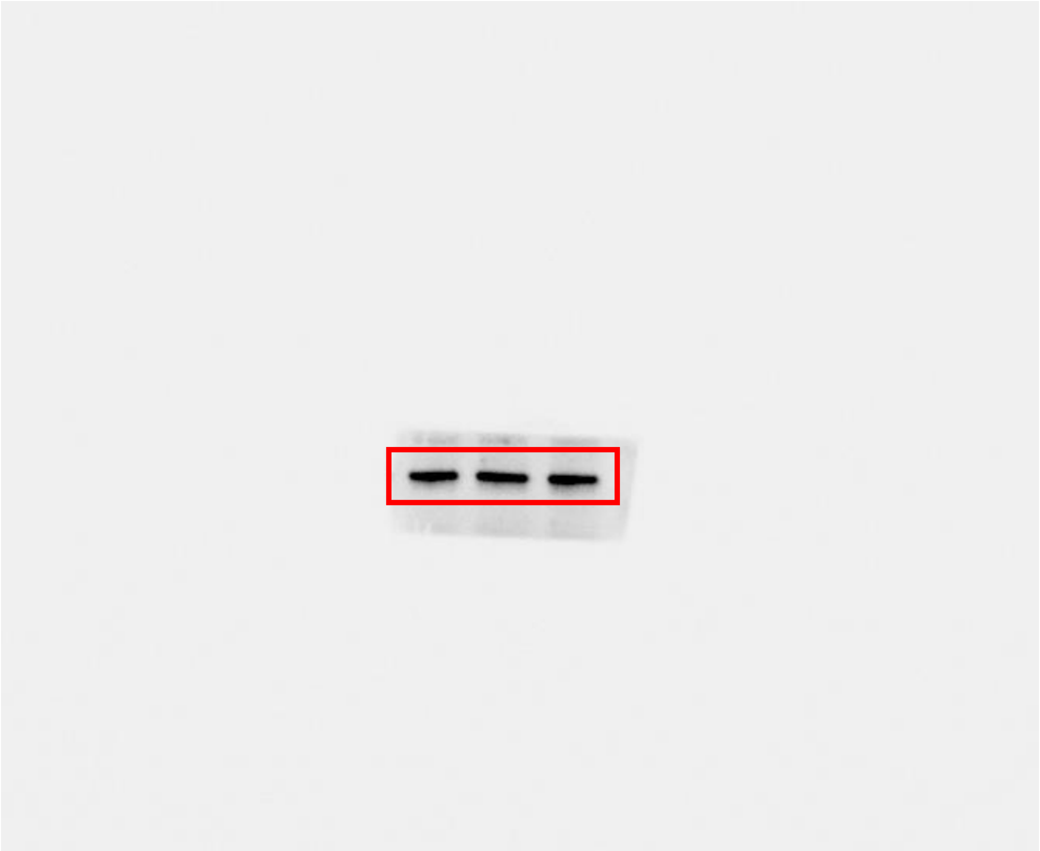

Figure S3F

TRIB3

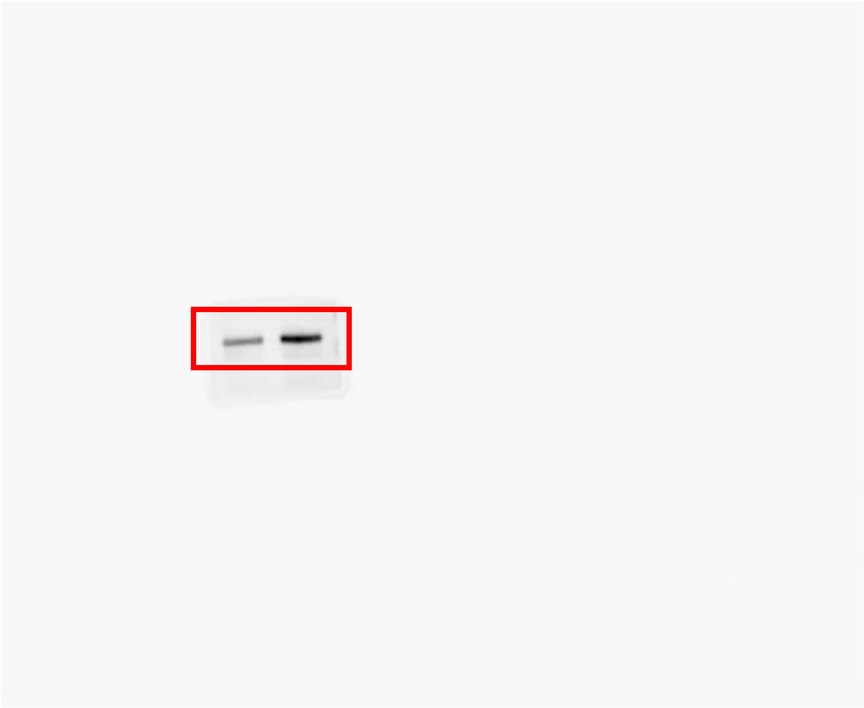

β-actin

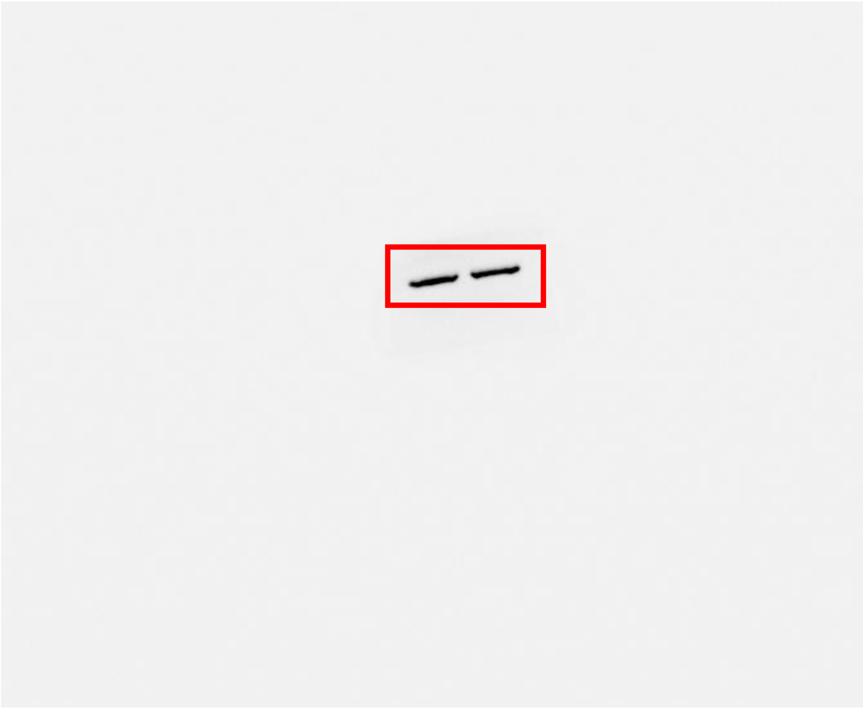

Supplement: Supplementary file 2 — western blot [file 41419_2024_7311_MOESM2_ESM.pdf]
